# Mosaic: Single-Cell Atlas of Stress

**DOI:** 10.3390/cells15090807

**Published:** 2026-04-29

**Authors:** Edward Siler Monk, Bianca Shieu, Dhruvita Kumbhani, Liang Fu, Albert Lin, Josephine A. Taverna, Carrie J. Braden, Charles Jeff Uribe-Lacy, Wensheng Zhang, Casey M. Sabbag, Tim H.-M. Huang, Sonya R. Hardin, Lixin Song, Chun-Liang Chen

**Affiliations:** 1 School of Nursing, The University of Texas at San Antonio, San Antonio, TX 78229, USA; monk@livemail.uthscsa.edu (E.S.M.); shieu@uthscsa.edu (B.S.);; 2 Graduate School of Biomedical Sciences, The University of Texas at San Antonio, San Antonio, TX 78229, USA; 3 Department of Molecular Medicine, The University of Texas at San Antonio, San Antonio, TX 78229, USA; 4 Health Science Center Libraries, The University of Texas at San Antonio, San Antonio, TX 78229, USA; 5 Metis Foundation, San Antonio, TX 78216, USA

**Keywords:** acute/chronic stress, hypothalamus–pituitary–adrenal axis, single-cell analysis, omics, cellular heterogeneity, transcriptomic landscape, biomarkers, depression, dementia

## Abstract

Stress has been prevalent and has become an epidemic health burden, loaded with chronic disorders. The stress response is an adaptive mechanism that prepares an individual to respond to threats or other stressors in a fight-or-flight situation. The stress response involves the induction of neurological and hormonal networks and is usually resolved when stress subsides; however, persistent stress leads to permanent and detrimental impacts on health. With the rise of advanced single-cell analysis technologies, a wave of basic and translational research aimed at elucidating stress has shed light on the underlying mechanisms. Among 80 studies in this review, stressors are classified into acute/chronic physical, physiological, and psychological groups, whereas some studies have more than one stress source. Single-cell RNA-seq was the dominant technology utilized in these studies. This advanced technique systematically reveals cellular heterogeneity in gene expression patterns and the differential transcriptomic landscape of stress response in a wide array of tissues and organ systems, e.g., the nervous system, the endocrine system, the immune system, and others. Bioinformatics identified a single-cell atlas of stress-specific cell subtypes, cell-to-cell interactions, and enriched pathways, showing promise for stress syndrome biomarkers, attenuation, and targeted therapy. The limits of these stress studies were mainly focused on transcriptomics, so future studies using multi-omics approaches across multiple organ systems will yield insights into stress disorders and novel therapeutic strategies.

## 1. Introduction

Stress prominently affects both society and individual health [1]. Exposure to stressors (e.g., adverse traumatic life events) is common during a lifetime, with an estimated 40–70% prevalence [2,3], and may lead to negative psychological consequences, depression, and anxiety [4,5,6]. Additionally, stress induces behavioral changes with increased smoking, substance abuse, accidents, insomnia, and eating disorders which facilitate a downward spiral of health [1]. Depressive disorders together exerted a taunting economic burden estimated to be $333.7 billion ($382.4 billion in 2023 US dollars), or $16,854 per adult in 2019 in the USA alone [7]. After the COVID-19 pandemic, a recent 2023 survey revealed an even more detrimental epidemiological landscape, with a quarter of adults (24%) in 2023 rating their stress levels as high, between 8 and 10. A rating of 1 indicated little or no stress, and 10 indicated a high level of stress [8]. For those aged 18 to 34, even more (34%) reported the same high stress level. The majority (58%) of those aged 35 to 44 reported chronic health conditions, while 45% were diagnosed with mental health illnesses. Understanding the underlying mechanisms of stress and related disorders would grant us leverage to counteract the pandemic of depression and anxiety disorders.

To that end, stress research has significantly advanced in theoretical development and experimental validations in the past eight decades. Homeostasis is a principal mechanism that life uses to maintain the internal milieu constant for survival [9]. Stress is an engineering term adopted by Hans Selye to model external or internal threats that challenge homeostasis [10]. The challenging threats to homeostasis are stimuli, also called stressors, that elicit stress responses in the human body upon appraisal of the stressors [11,12]. The stressors are physical (e.g., heat, cold, radiation, noise, pain, immobilization, and chemical stressors), physiological (toxic insults, an injury, and an infection), and psychological (anxiety, fear, frustration, marital separation, unemployment, and death), according to the sources [11,13]. Stress responses are a coping mechanism involving integrative neuroendocrine responses that condition individuals for fight-or-flight situations. However, conceptual frameworks and evidence showed that long-term repeated stress may overwhelm homeostasis and lead to psychopathological consequences (allostasis) [14,15,16,17].

Stress responses typically act along one of three primary pathways: the hypothalamus–pituitary–adrenal (HPA) axis, the sympathetic–adreno-medullar (SAM) axis, and the psychoneuroimmunological pathway (PNI) [18,19,20]. The SAM pathway is activated in response to physical stressors, whereas psychological stressors excite HPA signaling. After sensing a stressor, the SAM system activates the adrenal medulla and sympathetic nerves to release catecholamines (epinephrine and norepinephrine) that are circulated and elevated in the brain, smooth muscle, and other organs and bind to membrane-bound α- and β-adrenergic receptors [13]. Adrenergic receptors are G protein-coupled receptors, activating cyclic adenosine monophosphate (cAMP) signaling in the cells [18]. Activation of the HPA axis begins with the release of corticotropin-releasing hormone (CRH) from the paraventricular nucleus (PVN) of the hypothalamus. The CRH acts on its receptors, CRH-R1 and CRH-R2, and activated CRH-R1 in the anterior pituitary induces adrenocorticotropic hormone (ACTH) secretion. Circulating ACTH subsequently stimulates the adrenal cortex to release glucocorticoids and mineralocorticoids [20]. The stress-mediated PNI pathway is a bidirectional interaction of the nerve and immune systems through humoral (e.g., cytokines IL-6, TNF-α, and CRP), cellular (e.g., proinflammatory peripheral monocytes) and neural routes (e.g., afferent sensory nerves and vagus nerve) [19,21,22]. The released stress hormones, delivered to the whole body, immediately act on the nervous, endocrine, cardiovascular, respiratory, gastrointestinal, muscular, and reproductive systems [18].

The molecular and cellular mechanisms underlying stress have been a focus of intense research. The appraisal of stress initiates the expression of c-Fos, an immediate early transcription factor, in brain regions including the PVN of the hypothalamus to regulate CRH anabolism [23]. Global transcriptome studies revealed the abnormal transcriptional regulation and signaling pathway networks involved in psychiatric disorders, major depressive disorders, and posttraumatic stress disorders (PTSD) [24,25]. The upregulated *DUSP1* gene in dentate gyrus and CA1 pyramid cell layer of the hippocampus downregulates BDNF, VGF nerve growth factor, and VEGFA in the depression cohort [26]. Decreased synapse-related genes (*CALM2*, *SYN1*, *RAB3A*, *RAB4B*, and *TUBB4*) and loss of synapses in the prefrontal cortex (PFC) region are associated with depressive pathology [27]. A genome-wide association study identified 30 gene-based associations with PTSD in a large veteran cohort [28]. A meta-analysis of microarray-based studies showed that the stress hormone glucocorticoid regulates 88 genes, including growth factors, mTOR pathways and progression of cell cycle [29]. ChIP-seq identified 918 and 1450 nonoverlapping binding sites for mineralocorticoid receptor and glucocorticoid receptor (GR), respectively, while 475 loci were co-occupied by both receptors [30]. Under acute stress, the target genes include glucocorticoid-responsive genes FK506-binding protein 5 (*Fkbp5*), Period 1 (*Per1*), and serum- and glucocorticoid-inducible kinase 1 (*Sgk1*) [31]. However, differential genes associated with stress disorders might be obscured in these studies because they were mainly based on bulk tissues composed of heterogeneous cell types with diverse expression of stress hormone receptors (e.g., GR) and responses to stress hormones.

Single-cell analysis technologies have advanced tremendously in the last decade and have been demonstrated to be effective in stratifying cell subpopulations and revealing the cellular heterogeneity in preclinical animal models and clinical studies [32,33,34,35]. Initial success in single-cell RNA sequencing (scRNA-seq) has set the single-cell technical landmark, established its exponential growth, and paved the way to the development of single-cell genomics, epigenomics, proteomics, and multi-omics [36,37,38,39]. For epigenomics, a single-cell assay for transposase accessible chromatin (scATAC-seq) was invented to map open chromatin for the regulatory expression of genes [40]. Global DNA methylation heterogeneity for gene expression regulation can be evaluated using single-cell bisulfide conversion and genomic sequencing [39]. Multiplexing in single-cell proteomics was made possible by mass spectrometry or sequencing-based methods [41]. Single-cell omics have been applied to elucidate the underlying mechanisms of stress. Although some review papers have assessed the applications of omics to stress-related research, they focus either on bulk omics, stress impacts on a single organ/tissue (e.g., brain, hypothalamus, kidney) or pathophysiological perspectives without the stress role [24,25,33,42,43,44]. In this paper, we developed a single-cell-omics- and stress-centered strategy for the literature search. We reviewed recent advances and findings in stress research, leveraging single-cell techniques and bioinformatic methods to systematically map a single-cell atlas of stress.

## 2. Methods

**Figure 1:**
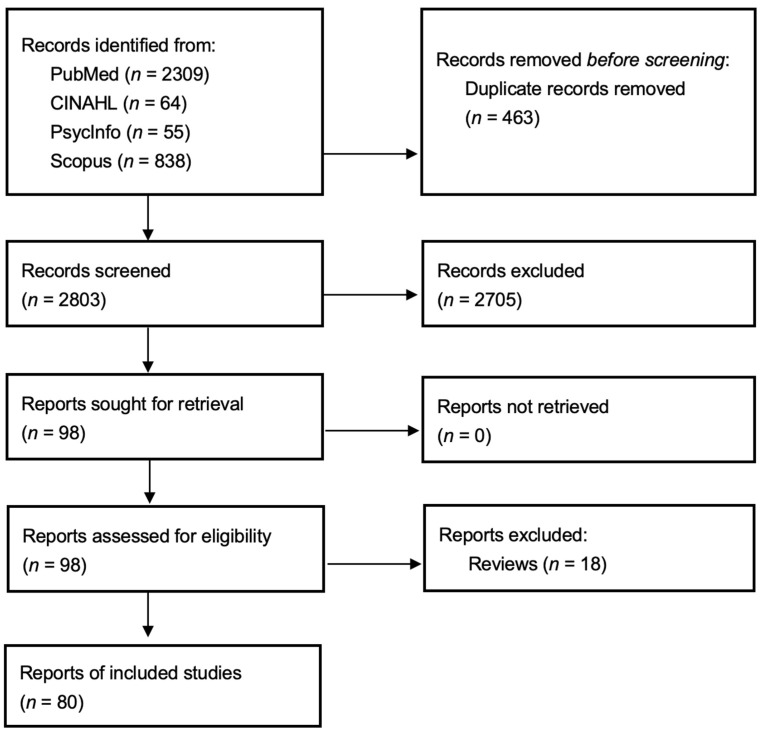
The flow chart illustrates the literature search strategy used in this review and the search outcomes.

Reports were included that focused on using single-cell omics to analyze the physiological impacts of stress, as shown in Figure 1. Reports were excluded if they contained no original extractable data, were not peer-reviewed research articles, were not about single-cell omics, focused solely on local stress at the molecular or cellular levels without considering systemic stress phenotypes and their correlations, or if the study focused on stress in plants or microorganisms.

Information sources were selected in consultation with a health sciences librarian. PubMed, CINAHL, PsycInfo via EBSCOhost, and Scopus were searched on 5 September 2023. The searches were repeated on 26 January 2026, to update the data before publication. In addition, the bibliographies from included reports were hand-searched for relevant reports.

Search strategies were developed in consultation with a health sciences librarian. To identify relevant reports, the search strategy incorporated terms related to single-cell omics and the physiological effects of stress. Appropriate subject headings were used in databases with controlled vocabularies. To improve relevance, search terms were field-restricted to subject headings, article titles, and abstracts, where permitted by the search options. No search limits were used. The first search strategy was reviewed by a second health sciences librarian using a modified PRESS checklist before being customized for the other databases [45,46]. The search strategy used for each database is documented in Supplementary Method S1.

Search results (*n* = 2803) from each information source (PubMed (*n* = 2309), CINAHL (*n* = 64), PsycInfo (*n* = 55), Scopus (*n* = 838)) were imported and collated into Endnote 20 (Clarivate, London, UK), which was used to automatically and manually remove duplicate records (*n* = 483). The records were provided to the research team as a .RIS file.

Two independent reviewers screened each record from a team of four according to the eligibility criteria described above. The full-text reports of the selected records were then screened according to the same criteria. Disagreements between reviewers during the screening process were reviewed and resolved by the senior author. A total of 80 reports were included for data extraction (Table 1).

## 3. Results

A total of 2803 articles were eligible for review. A total of 293 articles were excluded for reporting on the stress of plants and microorganisms. The remaining 2510 articles were then reviewed for titles, abstracts, and full texts. The review also filtered out reviews without detailed single-cell omics coverage or articles that focused solely on local stress at the molecular and cellular levels, without considering systemic stress phenotypes and their correlations. As shown in Table 1, the final filtration yielded 80 articles that displayed cellular response to variant stressors, including physical stress (*n* = 11), physical/psychological stress (14), physiological stress (37), physical/physiological stress (3), physiological/psychological stress (6), and psychological stress (9). These articles were published between 1996 and January 2026.

**Figure 2:**
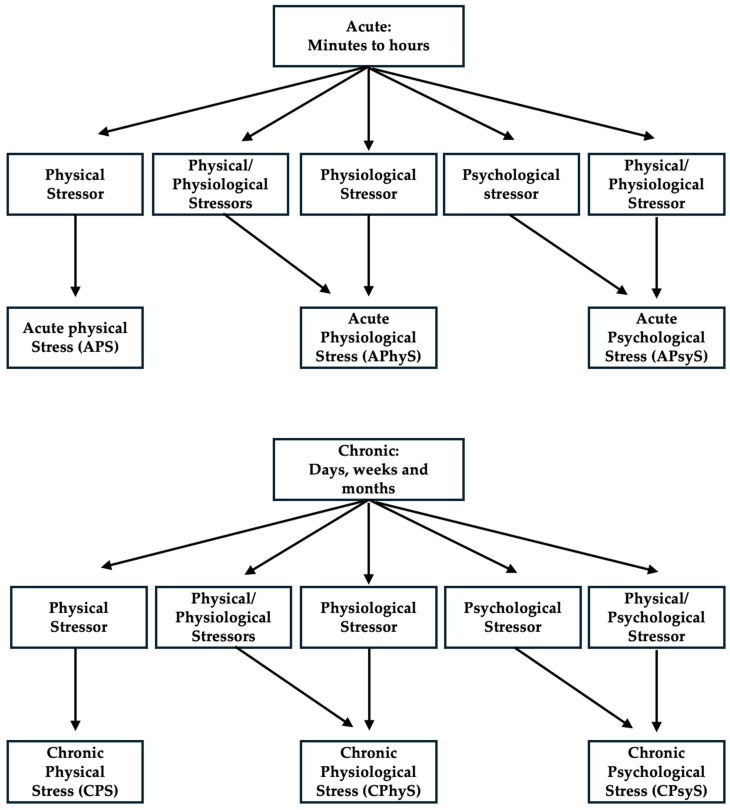
The schematic illustration of the six major categories of stress. There is acute physical, acute physiological, acute psychological, chronic physical, chronic physiological, and chronic psychological stress. The duration of stressors determines whether the stress is acute or chronic. The stress induced by the complex dual physical/physiological stressors is attributed to physiological stressors, given the relatively brief presence of physical stressors. The complex physical/psychological stressors are grouped into psychological stress due to traditional classification.

Different stressors elicit distinct patterns of stress responses (Figure 2). For exposure time, acute stress is defined as stress that lasts for minutes and hours, whereas chronic stress is persistent for hours per day for weeks or months [13,195]. Physical stressors are external stimuli that disrupt homeostasis; physiological stressors are derived from physiological challenges [196]; and psychological stressors are anticipated challenges, whether they will occur or not. Therefore, based on these definitions, the research articles were classified into six categories in the main text according to the stress duration and nature: (1) acute psychological stress (APsyS), (2) chronic psychological stress (CPsyS), (3) acute physical stress (APS), (4) chronic physical stress (CPS), (5) acute physiological stress (APhyS) and (6) chronic physiological stress (CPhyS). Some studies involved both physical and physiological stress, but they were assigned to the physiological group because physical stress was present for only a short time in Table 1. Some studies involved both physical and psychological stress, and we assigned them to the psychological stress category because these have long been adopted as psychological models.

## 4. Single Cell Technology

From the selected stress articles, single-cell RNA-seq (scRNA-seq) was the dominant single-cell technology utilized for cell gene expression profiling and subpopulation identification. In 2013, scRNA-seq was selected as the method of the year by *Nature Methods*. The scRNA-seq techniques have evolved with improved robustness and accessibility over the years. Among scRNA-seq platforms, the 10× Genomics Chromium platform has been widely used in stress studies, owing to its significantly increased throughput, single-cell oil-in-water emulsion droplets, high-density barcoding, and automation convenience. The second-most popular SMART-seq(1-2) methods offer the highest coverage of gene numbers and full length of genes as shown in the 7th column of Table 1 [196,197]. The rest of the single-cell analysis techniques were adopted in either one or two stress study papers. They are the Singleton GEXSCOPETM, CROP-seq, MARS-seq, STRT/C1, and CyTOF, among others. The big data analysis of scRNA-seq is reliant on bioinformatics methods that have also been advanced consistently in the last 15 years. The bioinformatics methods used in the reviewed papers are summarized in Table 1. CellRanger was the software widely used for the sequencing read alignment, filtration, barcode counting, and unique molecule index (UMI) counting, since the 10× Genomics Chromium platform carried out most of the scRNA-seq in these studies. The majority of gene count matrices were further analyzed using the Seurat suite for the identification of differentially expressed genes (DEGs) among treatments, data dimension reduction, and cell clustering [49,78,198]. Other commonly used dimension reduction, cell clustering, and cell pseudotime projection methods include Principal Component Analysis (PCA), t-distributed stochastic neighbor embedding (*t*-SNE), Uniform Manifold Approximation and Projection (UMAP), and Monocle [64,112,199]. The pathway analyses based on DEGs were mainly performed using Gene Set Enrichment Analysis (GSEA) and Ingenuity Pathway Analysis (IPA).

## 5. The Nervous System/The Endocrine System

**Figure 3:**
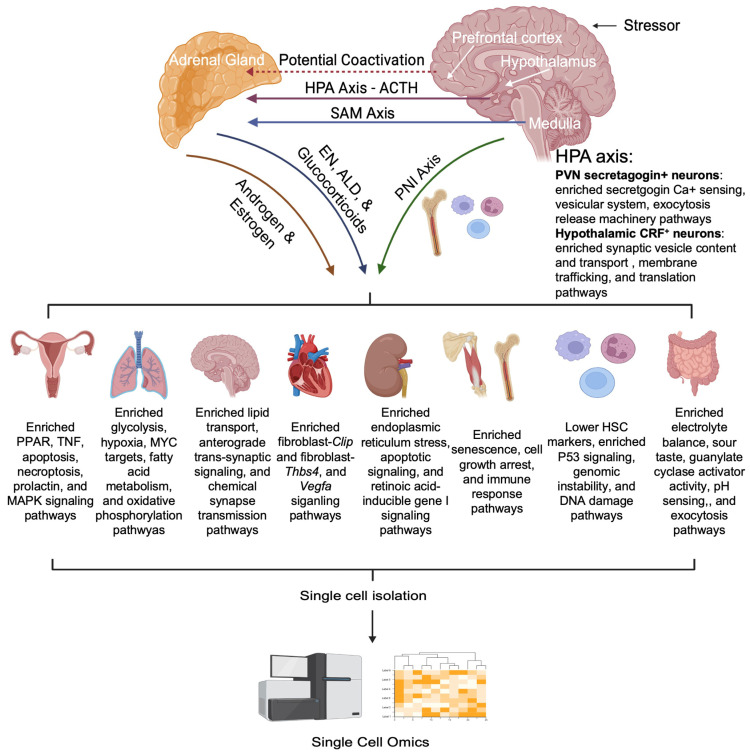
The scheme illustrates stressors, stress pathways, and their interactions with systemic organ systems. Arrows of the same color indicate related pathways. The impacts of stress on single cells isolated from multiple organs were subject to single-cell omics analysis. The representative enriched pathways of stress in organs are shown. ACTH: adrenocorticotropic hormone; ALD: aldosterone; HPA: hypothalamic–pituitary–adrenal; EN: epinephrine; HSC: hemopoietic stem cells; SAM: sympathetic–adreno-medullar; PNI: psychoneuroimmunological. Created with BioRender.com.

The brain processes stressors first, so the response to stress in the brain has been investigated by several research groups (Figure 3). Single-cell transcriptomic mapping reveals the variety of stress-related brain cells, including the paraventricular nucleus, the habenula, the amygdala, the hippocampus, the cerebral cortex, and nervous cell types. Most individuals are resistant to chronic stress and bypass stress-mediated mental dysfunction that some individuals are susceptible to. Molecular underpinnings for chronic stress vulnerability are still unavailable, and understanding the mechanisms holds promise for developing effective interventions. While corticotropin-releasing hormone (CRH) from parvocellular neurons in the hypothalamic PVN is the upstream regulator for subsequent hormone release, the precise molecular mechanism regulating its periodic release and the neural cell identities in PVN for that function are not well understood. After acute pain stress induced by subcutaneous formalin injection into the paws of mice, 192 cells of PVN were isolated using the C1-AutoPrep system (Fluidigm), single-cell capture, lysis, cDNA synthesis, and amplification for STRT/C1 scRNA-seq (APsyS) [34,131,198]. In PVN, oxytocin, arginine–vasopressin (AVP), somatostatin, Gamma-aminobutyric acid (GABA), and glutamate neurons displayed distinct transcriptomic profiles and clusters, while secretagogin^+^ neurons, as well as CRH^+^ and thyrotropin-releasing hormone (TRH)^+^ neurons, spread across clusters. From 132 cells’ transcriptomes and interference RNA experiments of secretagogin^+^ neurons from PVN, the team identified that a secretagogin locus of the mammalian hypothalamus controls stress hormone release [131,132]. Secretagogin^+^ neurons act through secretagogin’s Ca^2+^ sensing function, coordinating with the vesicular system and exocytosis release machineries to release the hypothalamic releasing hormone. The locus coeruleus (LC) is the major noradrenaline system source in response to arousal and stress stimuli, but how it regulates the process remains unclear (APS) [105]. SnRNA-seq and spatial transcriptomics of post-stress LC and peri-LC present a heterogeneous landscape of neural cell diversity, composed of three major groups (the glutamate, GABA, and monoamine neurotransmitters) and transcription patterns. Acute stress (restraint or forced swim test) induces anxiety-like phenotypes and increased expression of *c-Fos*^+^ in the somatostatin-positive cells of the S1 trunk region (APsyS) [162]. Forty-eight percent of the somatostatin-positive cells are GABAergic neurons that are likely the major responsive cells to acute stress.

Early-life adversity (ELA) advances vulnerability to stress and stress-related affective disorders, with mechanisms that have not been well defined. Hypothalamic corticotropin-releasing factor (CRF)-expressing neurons are potential candidate mediators for ELA because they respond to stress by regulating hormonal and behavioral changes. In the ELA mouse model, neonatal mice were subject to limited nesting and bedding materials simulating poverty until postnatal day 10–12 (CPsyS) [137]. Single-cell transcriptional changes in hypothalamic CRF-expressing neurons of both control and ELA mice identified eight clusters of cells, including five CRF+ clusters, microglia, astrocytes, or endothelial cells, according to cell identity marker genes. There were 46 DEGs identified between control and ELA CRF^+^ neurons, with 18 genes higher in ELA CRF^+^ neurons. These upregulated genes in ELA CRF^+^ neurons are heat-shock proteins (*Hspa8* and *Hsp90ab1*), *Psma6*, and *Ppia*, associated with response to environmental stressors. Some genes are involved in synaptic vesicle content and transport (*Vamp2*, *Chgb*, *Atp5a1*, and *Scn3b*), membrane trafficking (*Atp5a1*, *Vamp2*, and *Scn3b*), neuronal structure (*Actg1*, *Hspa8*, and *Stmn1*), and upregulation of translation (*Eif1*, *Mrps12*, *Rpl39*, and *Rps13*). Gene expression alterations were specific to glutamatergic subpopulations and involved in neuronal differentiation, synapse formation, energy metabolism, and cellular responses to stress and injury. The differential gene expression modifications were likely derived from adrenal hypertrophy and abnormal responses to stress in adulthood.

Habenula comprises lateral and medial components and is an anatomic hub regulating mood, fear, memory, and emotion. Using genome-wide gene expression analysis, a study identified 379 differentially expressed genes (DEGs) in the habenula of rats subjected to 14 days of chronic restraint stress (CPsyS) [176]. These DEGs are enriched in neuroactive ligand–receptor interaction, the cAMP (cyclic adenosine monophosphate) signaling pathway, circadian entrainment, and synaptic signaling from the Kyoto Encyclopedia of Genes and Genomes pathway analysis. They also respond to corticosteroids, positive regulation of lipid transport, anterograde trans-synaptic signaling, and chemical synapse transmission from the Gene Ontology analysis. Based on protein–protein interaction network analysis of the DEGs, neuroactive ligand–receptor interactions, circadian entrainment, and cholinergic synapse-related subclusters were identified. Additionally, cell type and habenular regional expression of DEGs, evaluated using a recently published single-cell RNA sequencing study (GSE137478), identified 26 cell clusters and assigned cell types. Enriched pathways from the KEGG and GO pathway analyses strongly suggest that DEGs related to neuroactive ligand–receptor interaction and trans-synaptic signaling are highly enriched in medial habenular neurons. These findings highlight the habenula’s role in the pathophysiology of stress-related disorders. Another group also showed how chronic mild stress (CMS) influences the habenula function at the single-cell level [54]. The same restraint stress model also showed impaired rhythmic gene expression in lacrimal glands, resulting in circadian decoupling of immune, metabolic, neural, and proliferative pathways and dry eye syndrome (CPsyS) [47]. The defects in circadian gene expression, glandular architectures, and tear secretion can be partially restored by propranolol or metyrapone. Mice subjected to an 8-week program of CMS displayed a deficit in sociability behavior, as the main phenotype of depression, and were suitable models for the study of depression (CPsyS) [54]. Single-cell transcriptomic profiling identified target genes that play a role in chronic stress, which induced depression and deficits in motivated behaviors. Firstly, the team developed an animal model to simulate chronic mild stress (CMS) and depression relation through a complicated scheme of controlled stress stimuli from the living environment. The CMS mice displayed anhedonia-related behavior which is the core symptom of depression, with a significant reduction in awarding preference in the sucrose preference test (SPT). The anatomical analysis demonstrated that CMS-induced higher burst and tonic firing in ventral tegmental area (VTA)-projecting lateral habenula (LHb) neurons are correlated with increased passive coping (PC) but not anxiety or anhedonia. ScRNA-seq analysis of limited neurons (*n* = 30 cells from 10 mice) indicated enrichment of synaptic glutamate receptors *Grik2* and *Grid2* genes and the neuronal excitability-relevant potassium channel *Kcnc2* gene in LHb → VTA compared to in LHb → DR (dorsal raphe) neurons. Moreover, there was a downregulation in gene expression for synaptic regulators *Lrrtm3* and glutamate-receptor subunit *Grin1*. When they identified differentially expressed genes in LHb → VTA neurons between depressed and control mice, the team found the upregulation of *Lrrtm3*, *Grin1*, and *Kcnc1* in the stressed counterparts.

The amygdala is the epicenter of fear and anxiety [200]. Evidence shows that immune molecules from immune cells elicit functional effects on the onset of anxiety-like behaviors, including IL-1b and IL-6 from innate immune cells and IFN-γ from the adaptive immune cells. However, there is a gap in understanding the pathological role and underlying regulatory mechanisms of the stress-induced peripheral T lymphocytes for mood disorders (CPsyS) [69]. Electronic foot shock (ES) was used to stress a mouse model of PTSD for 8 consecutive days to induce anxiety in control and recombination activating gene 1 (Rag1)^−/−^ mice. Control mice lost interest in exploring the central region and locomotion in the open-field test, while (Rag1)^−/−^ mice did not, indicating that T cells are involved in ES-related anxiety development. Metabolomes and single-cell transcriptomes of the amygdala have shed light on the activation of oligodendrocytes by xanthine derived from CD4^+^ T cells. The activation effects are apparent in genes of DNA synthesis and cell cycle pathways. Secondly, the upregulated purine is promoted by mitochondrial fission modulated by interferon regulatory factor 1 (IRF1) accumulation in CD4^+^ T cells.

Stress has an impact on the hippocampus, which is a critical brain region that responds to tone-induced fear reconsolidation [136]. Paired conditioned and unconditioned stimuli can induce contextual fear memory in Pavlovian classic fear conditioning. Neurons and glial cells in the hippocampus interact in reshaping fear memory-mediated synaptic plasticity through group II metabotropic glutamate (mGlu) receptors in astrocytes and inflammatory factors in microglia. However, the interactions of various cell types and corresponding global gene expression in fear remain unexplored. Single-cell molecular alterations (DEGs) in the hippocampus reveal target cells and pathways of conditioned fear memory (CFM) (APsyS) [136]. Data analysis discovered seven non-neuronal and eight neuronal cell clusters, including four newly identified neuronal subtypes. CA subtype 1 cell type expresses *Ttr* and *Ptgds* in response to acute stress and facilitates CFM. KEGG pathway enrichment shows pathways related to synaptic plasticity, virus infection, and inflammation (NF-κB and IL6) in hippocampal neurons and astrocytes in CFM reconsolidation. Long-term potentiation (LTP) pathway gene expression is upregulated in neurons (DG and CA1) but inhibited in astrocytes. A glial–neurovascular network was transcriptionally activated and provided resilience after chronic social defeat in a mouse model using in-depth single-cell transcriptomics (CPsyS) [150]. The experimental CSDS mice were classified into susceptible, intermediate, and resilient groups in contrast to the control group according to behavioral phenotypes and PCA stratification. Single-cell transcriptomic data collected from 29,358 dorsal and ventral hippocampal cells revealed 41 distinct clusters, including different cell types, e.g., astrocytes, neural stem cells, neurons, mural cells, endothelial cells, oligodendrocyte precursor cells (OPC), oligodendrocytes, and microglia. Further analysis indicated that the dorsal hippocampus in resilient mice carries out neuroimmune responses, angiogenesis, myelination, and neurogenesis, thereby enabling brain restoration and homeostasis in response to chronic stress. Molecular analysis suggests that the mTOR pathway plays a vital role in chronic response, and an inhibitor, rapamycin, was demonstrated to effectively target the pathway after stress and substantially improve stress resilience. The same model in another study demonstrated that CSDS-mediated circulating myeloid-derived MMP3 caused defects in the extracellular space and physiological functions in the nucleus accumbens and changes in social behavior (CPsyS) [50]. Deletion of MMP8 avoids these changes in neural anatomy and social behavior. Among the four cell clusters, previous microglia proinflammatory gene signatures were not present, possibly due to glial heterogeneity or scRNA-seq technology differences. Another line of evidence came from diabetes-associated physiological stress and undesired outcomes in the hippocampus (CPhyS) [108]. Diabetes-associated cognitive dysfunction (DCD) exhibits phenotypes similar to vascular dementia and Alzheimer’s Disease. How the impairment progression in cellular compartments of the hippocampus is connected with DCD has not been well defined. ScRNA-seq of hippocampus derived from db/db (4667 cells) and db/m mice (9954 cells) revealed 26 transcriptionally distinct clusters divided into 10 distinct cell types based on cell-type-specific gene expression [108]. The cell types annotated were B cells, endothelial cells, ependymal cells, fibroblasts, microglia, mural cells, neurons, NKT, oligodendrocytes, and oligodendrocyte precursor cells. The db/db mouse hippocampus suffered a dramatic decrease in the abundance of microglia and oligodendrocytes enriched with gene pathways of infection, inflammation, oxidative stress, and neurodegenerative diseases. The findings discovered potential diagnostic biomarkers and therapeutic interventions for DCD [108].

Multidisciplinary studies of PTSD and major depressive disorder (MDD) implicate the prefrontal cortex in disease risk and pathophysiology [201]. The single-cell atlas of the cerebral cortex under chronic psychological stress was explored in several independent studies. Stress vulnerability to chronic social defeat stress (CSDS) in the brain has been explored by electome factor 1 and scRNA-seq (CPsyS) [77]. Hundreds of DEGs were identified across five major cell types, with the majority of stress-vulnerability genes occurring in GABAergic neurons. Upregulated mitochondrial and metabolic pathways also contribute to stress vulnerability. Sox6^+^ interneurons are also involved in the development of MDD phenotypes in another CSDS mouse model (CPsyS) [109]. Weighted gene expression network analysis identified several gene modules related to inflammation, autophagy, and synaptic function. Chronic stress induces microglial-mediated inflammatory responses and compromises oligodendroglial and neuronal homeostasis in the prefrontal cortex, leading to depression (CPsyS) [87]. CPsyS can induce microglial inflammatory reactions, leading to oligodendroglial-lineage (OLN) and neuronal deficits in a mouse model for depression. The Repeated Social Defeat Stress (RSDS) paradigm was used to study chronic stress’s early and late effects in adult male mice. Adult male mice were subjected to repeated physical aggression (‘‘defeats’’) from aggressive CD-1 mice for 10 consecutive days. Significant inflammation was observed in prefrontal and ventral/lateral cortical areas, including recruitment of activated proinflammatory microglia, increased phagocytosis, oxidative stress production, and proinflammatory cytokine release in the stress group. Maternal separation and social isolation have alleviated anxious behaviors and the expression of stress-related corticotropin-releasing hormone receptor 1, sex-determining region Y-box 2, and doublecortin. Early adversity stress also affects social subordination behavior in the mouse model (CPsyS) [89]. Exposure to acute social stress in the tube test excites upregulation of cell type-specific alternations of the single-cell transcriptomc landscape in glutamatergic and GABAergic neurons in the ventral hippocampus of ELA mice. The ELA-induced social subordination can be corrected in early sensitive stages by enhancing the inhibitory network function using transient diazepam treatment. In a double-hit mouse model, mice subject to maternal infection stress and RSDS presented increased microglia in the cerebellum (CPsyS) [72]. Single-cell proteomics identified 38.4% loss of Purkinje cells in lobule VIa-VIb and a microglia cell transition to *TREM2*^+^ stress-associated microglia characterized by elevated IL-6 and TGF-β signaling in the double-hit cerebellum. The data indicated stress synergy in phenotypic and molecular reshaping in the cerebellum. From prefrontal cortex scRNA-seq of MDD patients and controls, the distinct clusters were labeled based on their expression of oligodendrocyte progenitor cells (OPC) and Committed OPC (*PCDH15*, *DSCAM*, *VCAN*, *SOX6*, *PDGFRα*, *CSPG4*, *OLIG1*, *OLIG2*), as well as Immature Pre-myelinating and Mature Myelinating Oligodendrocyte (*CNTNAP2*, *CLDN11*, *CNP*, *PLP1*, *PCDH9*, *QKI*, *MBP*, *MOG*, *MAG*) markers [88]. The one unique MDD cluster was labeled as Immune Oligodendrocytes (Im-OL), based on their expression of immune markers (*P2RY12*, *CD74*, *C3*, *ITGAX*, *ITPR2*, *ARHGAP24*, *ADAM28*, *LPAR6*) with immune features and myelination deficits. Transcriptional profiling of 32 (dorsolateral pre-frontal cortex) DLPFC samples at bulk or single cells from 11 human patients with PTSD, 10 with MDD, and 11 control subjects was conducted to identify pathological DEGs and deregulatory pathways (∼415 K nuclei; >13 K cells per sample) (CPsyS) [58]. Twenty-two cell clusters were discovered among eight major cell types arranged sequentially from the largest to the smallest: excitatory and inhibitory neurons, oligodendrocytes, astrocytes, OPCs, microglia, endothelial cells, and pericytes. The analysis of 194 DEGs indicated that 38 were associated with PTSD and 156 genes with MDD, mainly in excitatory neurons with 89% and 87%, respectively. Enrichment analysis revealed significant enrichment pathways in astrocytes, excitatory, and inhibitory neurons. In a combined larger cohort of PTSD and MDD patients, snRNA-seq data of 510 K cells showed eight major cell subtypes (CPsyS) [67]. Statistically significant 52 DEGs were present in PTSD patients and 779 DEGs in MDD patients, with 716 DEGs newly reported. Epigenetic regulation of gene expression of MDD was explored in the prefrontal cortex of 44 patients and 40 control samples using a single-nucleus assay of transposase-accessible chromatin (snATAC-seq) (CPsyS) [59]. From 201,456 high-quality nuclei, seven cell types and 38 distinct clusters were identified. About 76% of differentially accessible regions were less accessible, mainly in MDD patients’ microglial and external excitatory neuron clusters. A multi-omics analysis of 111 dorsal-lateral prefrontal cortex samples (36 PTSD, 36 MDD, and 39 controls) uncovered single-cell-type gene expression, regulatory changes, and cell-to-cell interactions that underlie PTSD pathology (CPsyS) [82]. Seven major cell types (excitatory neurons, inhibitory neurons, oligodendrocytes, oligodendrocyte progenitor cells, endothelial cells, astrocytes, and microglia) and 61 subclusters were identified. The team found 322 DEGs in excitatory neurons, 66 in inhibitory neurons, and 1184 unique genes across seven major cell types. DEGs were enriched in the pathways of ubiquitin binding, cell stress response, and cadherin binding. Among DEGs, CTNNA3 and HSPA1A were upregulated in PTSD patients but downregulated in MDD counterparts. The snRNA-seq findings were further confirmed by spatial transcriptomes through the gene expression correlation analysis.

With a similar foot shock model (described above) and contextual fear treatment, Li et al. revealed that the loss or suppression of microglia in the corticolimbic system alleviates behavior of PTSD in mice (CPsyS) [93]. Two rounds of foot shocks induced PTSD-like fear response on days 3, 8, and 15. To study the changes in immune cell landscape in the brains with PTSD, the immune cells were isolated and profiled by single-cell proteomic cytometry by time-of-flight (CyTOF). The number of microglia, the primary immune cell type, and the ratio of microglia to immunocytes were significantly increased in the prefrontal cortex (PFC) and hippocampus (HP), but not in the amygdala (AMY), on the fifth day of foot-shock exposure. Genetic/pharmacological depletion of microglia or inhibition of microglia activation alleviated PTSD-like symptoms in mice. The data indicated that microglia are the major brain immune cells in response to PTSD and pose a potential PTSD therapeutic target. Accumulating evidence suggests that reactive astrocytes are involved in multiple neurological pathologies and stress in the brain [202,203]. A large range of single-cell transcriptomics, spatial transcriptomics, and proteomic datasets were collectively integrated for the mechanistic role of astrocytes for Alzheimer’s disease (AD), Parkinson’s disease (PD), Huntington’s disease (HD), multiple sclerosis (MS), epilepsy (Epi), and chronic traumatic encephalopathy (CTE) (CPsyS) [121]. From nearly 170,000 astrocytes derived from 302 samples of human cortex (CTX), hippocampus (HIP), white matter (WM), and basal ganglia (BG), astrocytes characterized with high *ADGRV1*, *AQP4*, *SLC1A2*, and *GFAP* gene expression were classified into eight transcriptionally unique subpopulations. They identified clusters 0 and 1, both highly expressed homeostasis signatures suggesting functions related to amino acid transport, transportation across the blood–brain barrier, and synaptic transmission (e.g., *SLC1A2*, *SLC1A3*, and *GLUL*). Cluster 2 demonstrated highly expressed genes involved in oxidative stress (*FOS*, *JUN*/*JUNB*, *UBC*, and *ID3*) and the extracellular matrix (*TNC*, *TNR*, and *VCAN*) and associated with proteostasis (e.g., *HSP90AA1*, *HSPA1A*, and *HSPB1*, known as chaperones). The team also found seven gene modules implicated in disease onset and progression. The scRNA-seq of Alzheimer’s disease (AD) patients’ posthumous brain tissues analyzed 80,660 single-nucleus transcriptomes from the prefrontal cortex of 24 individuals with high levels of β-amyloid and varying degrees of AD hallmarks and 24 controls with no pathology (CPsyS) [110]. Gene expression levels in the prefrontal cortex (Brodmann area 10) identified the major cell types: excitatory neurons (marked by *NRGN*), inhibitory neurons (*GAD1*), astrocytes (*AQP4*), oligodendrocytes (*MBP*), microglia (*CSF1R* and *CD74*), oligodendrocyte progenitor cells (*VCAN*), endothelial cells (*FLT1*), and pericytes (*AMBP*). In the comparison of gene expression between pathology versus non-pathology individuals, the team identified 1031 DEGs that are mainly downregulated in excitatory or inhibitory neurons and upregulated in oligodendrocytes, astrocytes, and microglia. The transcriptionally distinct subpopulations include those associated with pathology and are characterized by myelination, inflammation, and neuron survival regulators. The strongest disease-associated changes appeared early in pathological progression. They were highly cell-type specific, whereas genes upregulated at late stages were common across cell types and primarily involved in the global stress response. In an AD progression mouse model, a terminally exhausted inflammatory microglial subpopulation with inflammatory signals and cell-intrinsic stress markers was noted (CPsyS) [118]. It is concomitant with a high *APOE4* expression that was rarely found in 10~22-week-old mice but exponentially expanded in 96-week-old mouse brains, suggesting APOE4’s role in AD progression and therapeutic targeting.

Microglia play a role in inflammatory responses during PD development and the progression of neurodegeneration. Mutations of the Parkinson’s disease gene *LRRK2* encoding Leucine-rich repeat kinase induce a dominantly inherited form of PD, and variations in the LRRK2 locus are the risk markers for PD development. The relationship between the *LRRK2* gene and microglia is still largely unclear. In order to validate *LRRK2* function in activation of microglia induced by intrastriatal injections of lipopolysaccharide (LPS) as a general inflammatory insult, single-cell transcriptomes of mouse brain microglia cells showed microglial activation DEGs as compared with control mice injected with PBS (CPsyS) [134]. From 705 cells from PBS-treated animals and 3005 cells from LPS-stimulated animals, increased *Sod2* and decreased *Txnip* were associated with active microglia. Moreover, *Irg1*, *NFKBiz*, *Saa3*, and *Cd83* were involved in microglia activation. A post-stroke depression rat model with unilateral middle cerebral artery occlusion and chronic unpredictable mild stress (CUMS) demonstrated enriched endothelium and microglia and impaired oligodendrocytes in the hippocampus, compared to the other three control groups (CPsyS) [94]. The scRNA-seq data identified 17 clusters, including endothelium, oligodendrocytes, microglia, pericytes, T/NK cells, and B cells. The data suggested a strengthened blood–brain barrier in the dual-hit rats.

Several studies focused on the impacts of CPhyS on the nervous system. Previous studies indicated that glioma and ischemic cerebral infarction share common pathways, suggesting the crucial roles of hypoxia and RNA-binding proteins (RBPS) in their pathological development (CPhyS) [103]. First, a panel of RBPS (*POLR2F*, *DYNC1H1*, *SMAD9*, *TRIM21*, *BRCA1,* and *ERI1*) and other parameters was developed as an effective predictor for glioma patients’ overall survival in the TCGA glioma cohort and an MRI-radiomics classifier. ScRNA-seq of normoxia and hypoxia cerebral cortex and UMAP depicted 16 cell clusters with differential expression of RBPS among the groups. *Irf5*/*Trim21* and *Tcf712*/*Brca1* in microglia, *Tcf712*/*Brca1* in astrocytes, and *Taf7*/*Trim21* in pericytes were responsible for hypoxia-induced regulation and phenotype. The understanding of neuronal response to oxidative stress, commonly implicated in neurodegenerative disorders, is crucial for elucidating gene functions in neurodegeneration and uncovering the mechanisms of disease. A sophisticated CRISPRi/a model and single-cell CROP-seq were used to systematically target oxidative stress response genes and pathways and revealed an unexpected function of *Psap*, encoding prosapsin, in response to removing antioxidants from cultured iPSC-derived neurons (CPhyS) [146,147]. The deficiency of *Psap* induced the formation of lipofuscin, a hallmark of aging, and generated reactive oxygen species and ferroptosis. The *Psap* loss-of-function phenotypes were only observed specifically in neurons, but not in the other cell types. Alpha-synuclein (SNCA)-A53T mutation discovery is the first genetic evidence for human PD. Induced pluripotent stem cell (iPSC)-derived neurons have provided profound improvement for in vitro study models of dopamine neuron-specific stress responses in human PD (APhyS) [70]. Here, the group performed a large single-cell transcriptomic study of human iPSC-derived dopaminergic neurons to elucidate gene expression dynamics and cellular heterogeneity in response to oxidative and genetic stressors (rotenone and tunicamycin). The group identified multiple neuronal subtypes with transcriptionally distinct profiles and differential sensitivity to stress, highlighting cellular heterogeneity in dopamine in vitro models. They also validated robust expression of PD Genome-wide associated study (GWAS) genes and overlap with postmortem adult substantia nigra neurons. Importantly, stress signatures were ameliorated using FDA-approved felodipine. Using isogenic SNCA-A53T mutants, they found perturbations in glycolysis, cholesterol metabolism, synaptic signaling, and ubiquitin-proteasomal degradation. Overall, the single-cell study furthered our understanding of PD and implicated the potential of cell replacement therapies. PD is a neurodegenerative disorder that exhibits motor symptoms and nonmotor symptoms, including cognitive loss, anxiety, and depression, affecting 6 million people in the world [204]. Access to human PD brain tissues is limited, so the iPSC-induced neurons have become a valuable model for interrogating the disease progression. Using an initial Principal Component Analysis (PCA), single-cell sequencing of 146 pure iPSC-dopamine neurons derived from a patient with more advanced PD distinguished these neurons from two PD patients with early pathology and three controls (CPhyS) [90]. iPSC-dopamine neurons have 143 over-dispersed genes, identifying the signal recognition particle (SRP)-dependent co-translational protein targeting the membrane pathway. Bayesian transcriptomic trajectories and pseudotime axis reconstructed the disease progression of PD, leading to endoplasmic reticulum stress, and identified HDAC4 as a regulator of Parkinson cell phenotypes. Cellular senescence is a complex stress response characterized by four hallmarks: cell cycle arrest, macromolecular damage, a secretory phenotype, and deregulated metabolism. Accumulation of cellular senescence may lead to various diseases. A study provided a comprehensive analysis of the expression and co-regulation of senescence genes (SnGs) across various human tissues and cell types (CPhyS) [165]. Using integrative gene network analysis of bulk and scRNA sequencing data from non-diseased human tissues, including the brain, the researchers identified SnG-enriched gene modules and characterized their co-expression patterns. They discovered 51 highly conserved SnGs, including key regulators like CDKN1A (p21), which control cell cycle progression and the senescence-associated secretory phenotype (SASP). The study highlights the remarkable cell-type specificity of SnG signatures, particularly in fibroblasts, endothelial cells, and immune cells. It provides a blueprint for future research on senescent cells and their interactions in human tissues.

## 6. The Immune System

Studies have shown that stress impacts the immune system and, depending on the duration and intensity, can lead to immunoprotective, immunopathological, or immunoregulatory/inhibitory responses [205]. Stress induces neuroendocrine secretion of adrenocorticotropin, prolactin, growth hormone, catecholamines, epinephrine and norepinephrine from HPA axis in the brain, adrenal gland and sympathetic nervous system [206]. A variety of immune cells express glucocorticoid receptors and adrenergic receptors to respond to stress hormones and elicit the activation of immune cells and cytokine production as an immune response (Figure 3) [206,207]. Chronic stress has a detrimental effect on the immune system, as exhibited in reduced lymphocytes, NK cell activity, helper/suppressor ratio, and antibodies. Several studies have focused on interrogating global transcriptomes of peripheral blood mononuclear cells (PBMCs) in response to stressors such as smoking, nanoparticles, chemoagents, and diseases. Hematopoietic stem cells (HSCs) are a subpopulation of pluripotent cells residing in bone marrow. Not only do they self-renew themselves but also generate all immune cell lineages to maintain the normal function of the immune system. Understanding the stress effects on this group of cells is of particular interest. A study investigated the role of the Phf6 gene in HSC aging (CPhyS) [157]. The team performed scRNA-seq analysis of LSK (Lin^−^Sca-1^+^CD117^+^) cells, consisting of hematopoietic stem and progenitor cells derived from the bone marrow of both young adult (16-week-old) and aged (24-month-old) wild-type and hematopoietic-specific Phf6-knockout mice. They identified 10 robust cell clusters with distinct transcriptomic patterns, in which the HSC cluster displayed long-term and short-term markers of HSC and low expression of immune cell lineage markers. The aged adult HSC showed the expansion of total HSC populations and decreased proportions of multipotent progenitor cell group 2-4 (MPP2-4), as compared to young adult counterparts, but the defective phenotypes were largely rescued by Phf6-KO. The researchers found that long-term HSCs from aged Phf6-knockout mice exhibited significant epigenetic changes and transcriptional programs that reduced genotoxic stress-induced aging. This suggests that Phf6 is a crucial epigenetic regulator of HSC aging, and its inactivation can potentially reverse age-associated functional decline in these cells. Genomic instability and DNA damage play a role in the aging of HSCs. Little is known about the endogenous causes and physiological progression of genomic instability of aged HSCs. Previous studies showed that genotoxic aldehydes in HSCs cause accelerated aging and myeloid bias (CPhyS) [153]. To investigate the molecular impact of *Aldh2* and *Fancd2* deficiency on HSC aging, scRNA-seq was applied to transcriptomic profiling of Lineage^−^ [Lin^−^] c-Kit^+^ Sca-1^+^ (LKS) HSCs of 8- and 12-week-old Aldh2^−/−^ Fancd2^−/−^ mice. The enrichment of Gene Ontology term analysis stressed that p53 signaling pathway (*Cdkn1a*, *Pmaip1*, *Ccnd1*, *Gtse1*, *Phlda3*, *Zmat3*, *Plk2*, *Sulf2*, *Epha2*, *Rps27l*, *Gdf15*, and *Eda2r*) and aging signature genes (*Selp*, *Mt1*, *Gstm2*, *Clca3a1*, *Cd38*, and *Neo1*) were associated with aging in Aldh2^−/−^ Fancd2^−/−^ HSPCs. The deletion of p53 rescued the aged Aldh2^−/−^ Fancd2^−/−^ HSPCs phenotype, with increased epigenetic age, telomere attrition, and myeloid-biased differentiation quantified by single-HSC transplantation. The data demonstrated that metabolism-derived formaldehyde-DNA damage stimulates the p53 response in HSCs to drive accelerated aging. A wealth of evidence supports the fact that HSCs maintain self-renewal and survival using metabolic and proteostatic regulation through the pro-survival integrated stress response (ISR) toward stressors derived from xenotransplantation and ex vivo culture for expansion. The study by Xie et al. (2019) investigated how sphingolipid metabolism influences the self-renewal of human HSCs (CPhyS) [163]. The researchers discovered that modulating sphingolipid levels, particularly through inhibition of the enzyme DEGS1 and potent bioactive lipid sphingosine-1-phosphate (S1P) by the synthetic retinoid fenretinide/N-(4-hydroxyphenyl) retinamide (4HPR), is crucial for maintaining HSC function and preventing the commitment to the hemopoietic stem and progenitor cell (HSPC) lineage. After treatment of 4HPC, HSC displayed a strong cellular stress theme in pathway analysis, including ER stress/UPR/ATF4, protein folding, ROS, and autophagy. This modulation helps preserve HSC self-renewal by reducing cellular stress and promoting metabolic quiescence. In scRNA-seq data analysis of long-term (LT) HSC, a cell-cycle-primed cluster and another non-primed cluster were identified. The primed cluster had higher CD38 surface expression, cell cycle programs, and CDK6 and DEGS1 expression than non-primed LT-HSCs. The findings suggest potential stress strategies for enhancing HSC quality and expanding their numbers for clinical applications. The article by Yu et al. delves into the intrinsic variability of HSCs and the role of epigenetic memory in driving this heterogeneity (CPhyS) [179]. Employing the Cre-LoxP system to generate HSC clones by multiple combinations of fluorescent markers, as the Hue mouse multi-colored tagging technique, the authors demonstrated the presence of highly persistent and fluctuating heterogeneous clones of HSCs under normal conditions, LPS stress, and irradiation stress. Based on single-cell transcriptomics and epigenetic profiling, the study reveals that HSCs exhibit distinct transcriptional, DNA methylation, and chromatin accessibility patterns, which are conserved even under stress conditions such as transplantation and inflammation. These findings suggest that the diverse behaviors of HSCs are largely governed by epigenetic configurations rather than transcriptional states alone. This research highlights the need to refine our understanding of stem cell plasticity and the stem cell niche, emphasizing the importance of epigenetically driven cell autonomy in hematopoiesis. Evidence indicates that inflammatory stress induces disproportional clonal expansion of Dnmt3a-mutant HSCs and malignant development. The molecular causes underlying the HSC fitness of resistance to inflammatory stress are still understudied. In order to study the mechanisms of Dnmt3a mutations, interferon gamma (IFNγ) was used as a hematopoietic stress model (APhyS) [194]. Bone marrow cells from control and Dnmt3a^KO^ groups were treated with either PBS or acute IFNγ inflammatory stress twice in 24 h before being isolated for scRNA-seq. All the single cells were clustered into two groups based on 50 DEGs and the treatment division. Dnmt3a-mutant HSCs overcame IFNγ-mediated depletion by overexpression of *Txnip* by DNA hypomethylation, leading to p53 stabilization and p21 upregulation. IFNγ-signaling is required for clonal expansion of Dnmt3a-mutant HSCs. The mechanism renders Dnmt3a-mutant HSCs’ increased quiescence and resistance to IFNγ-induced apoptosis. Accumulative evidence indicates that mature myeloid immune cells and HSPCs in bone marrow are the responders to bacterial invasion. How the dangerous stress of microbial infection is translated into the immune response of HSPCs, manifested as hemopoiesis, remains unclear. Sophisticate mouse models with a single-cell proteomics platform demonstrated that LSK cells (defined as Lineage−Sca1^+^cKit^+^), a mixture with long-term HSCs (LT-HSC), short-term HSCs (ST-HSC), multipotent progenitor cells (MPPs), and lymphoid-biased MPPs (LMPPs), secrete abundant, diverse cytokines through nuclear factor κB (NF-κB) signaling in response to the LPS-Toll-like receptor 4 ligand (TLR-4) and Pam3CSK4-TLR-2 stimulation (APhyS) [208]. Approximately 12.9% and 37.9% of LSK cells produced cytokines in response to LPS and Pam3CSK4, respectively. Among a panel of 12 cytokines, IL-2, IL-4, IL-17 and IFN-γ were mainly found in lymphocytes, whereas IL-2, IL-4, IL-17, and IFN-γ were generated in myelocytes. An unsupervised clustering of a single-cell proteomics study identified two distinct groups (i and ii) with variant distinct secretory cytokine profiles. Group i, resembling myeloid cells, expressed IL-6, TNF-α, IL-12, and GM-CSF, while group ii appeared to be lymphocytes generating IL-2, IL-4, IL-10, and IFN-γ. Upon further analysis, group i contained non-producers (subset 1) and group ii had super-producers (subset 4). Principal Component Analysis of single-cell proteomics revealed high heterogeneity in cytokine profiles within the four subpopulations of LSK cells which are solely dependent on cell surface markers. The classification of heterogeneous LSK cells deserves more detailed future investigation. The complexities and mechanisms of the bone marrow microenvironment regulating hemopoiesis and response to stress have been incompletely elucidated. In an acute bone marrow stress mouse model using the peritoneal injection of a chemotherapeutic agent, fluorouracil (5-FU), scRNA-seq was employed to stratify cell subpopulations of bone marrow VE-Cad^+^, LEPR^+,^ and COL2.3^+^ cells (APhyS) [149]. Cell clustering analysis discovered two subpopulations for endothelial Cad^+^ cells (V1-2), four subpopulations for perivascular LEPR^+^ cells (P1-4), three osteo-like COL2.3^+^ subpopulations (O1-3), and a small proliferating cell group (C). In response to acute stress, bone marrow exhibited a phenotype with decreased cell counts in Lineage (Lin)−SCA-1^+^CD117^+^ (LSK) bone marrow cells, vascular, and perivascular cell populations. Meanwhile, upregulation of adipogenesis-associated pathways and decreased expression of osteo-lineage-related genes were observed.

Stress reshapes the transcriptomic landscapes of innate immune cells and T cells in the thymus. The molecular causes of autoimmune central nervous system (CNS) degeneration diseases are largely unknown. Single-cell transcriptional profiling of oxidative-stress-inducing ROS^+^ innate immune cells from the spinal cord revealed the mechanisms underlying oxidative stress in neuroinflammation and promising therapeutic targeting (CPhyS) [117]. An autoimmune animal model used to recapitulate multiple sclerosis (MS) with paralysis and inflammatory demyelination, a symptom of chronic experimental autoimmune encephalitis (EAE), was induced in C57Bl/6 mice by the epitope of amino acids 35–55 of myelin oligodendrocyte glycoprotein (MOG) (‘MOG_35–55_ EAE’). In CNS ROS^+^ innate immune cells, the transcriptomic signatures showed coexpression of oxidative stress, coagulation and glutathione-pathway genes in an oxidative-stress-activated microglia cluster and infiltrating macrophages. An anti-cancer drug, activicin, remarkably reduced oxidative stress markers, proinflammatory gene expression, demyelination, neuronal damage, microglia activation, and macrophage infiltration in spinal cord lesions in MOG_35–55_ EAE mice. Acute myeloid leukemia exhibits a strong stress-induced suppression phenotype of natural killer (NK) cells with malignant infiltration of proliferative clonal immature myeloid cells in bone marrow. Single-cell RNA-seq of Lin^−^CD56^+^ NK cells from 8 healthy and 8 AML individuals discovered the stress effects of AML on NK cells (CPhyS) [63]. Based on differential gene expression of 23,000 NK cells, NK cells from healthy individuals were unsupervised and clustered into four subpopulations, including hNK-Bm1-4. hNK-Bm4 represented adaptive NK cells with NKG2C+ expression, found only in three individuals. hNK-Bm1, 2, and 3 belong to canonical NK cells that are negative for NKG2C^+^ expression. The pseudotime algorithm Monocle DDRTree predicted developmental trajectories, indicating that hNK-Bm3 (NK0) may act as a precursor and differentiate into hNK-Bm1 (NK1-CD56^dim^) and hNK-Bm2 (NK2-CD56^bright^) subsets. As compared with AML NK cells, NK cells from healthy donors have 90 upregulated genes and 107 downregulated genes. Bone marrow NK cells from AML patients have higher expression of interferon-induced genes (*IFI44L*, *IFI6*, *IFIT3*, and *IFI44*), HLA molecule-encoding genes (*HLA*-*DPB1*, *HLA-DPA1*, *HLA-DRB5*, and *HLA-DRB1*), *ZEB2*, and *KLF2* that regulate NK cell maturation and survival. The pathway gene enrichment analysis indicated that NK cells from AML individuals have dominant “response to cytokine” and “type I interferon signaling pathways” signatures. Hypertension is commonly found in 30% of the population, with increased morbidity and mortality. Cardiomyocytes of adults are not proliferative and respond to hypertensive stress with cellular enlargement to attenuate wall stress and maintain contractile function (A/CPS) [184]. Cardiac macrophages were generally classified into two groups: self-renewing and monocyte-dependent macrophages. Monocyte-dependent macrophages have been known for promoting tissue damage and fibrosis under hypertension, but little is known about the role of self-renewing macrophages. In normotensive mice, single-cell RNA-seq of fate-mapped self-renewing cardiac resident macrophages (RMs) showed heterogeneous cell states with distinct transcriptomes that were composed of a core repertoire of reparative gene programs with high insulin-like growth factor-1 (*Igf1*) expression [184]. In an established angiotensin II-induced acute, subacute, and chronic hypertension model, cardiac hypertension facilitated in situ proliferation and some cardiac RM states with transcriptional activation, associated with increased cardiomyocyte mass. RM transcriptomics exhibited four distinct clusters, with one cluster composed of the majority of proliferative RM at day 4 hypertension and nine transcriptomic states. During hypertension, targeted removal of RMs or knockout of RM-derived Igf1 prevented adaptive cardiomyocyte growth, leading to ultimate cardiac dysfunction. Single-cell transcriptomics indicated the adaptive role of a conserved IGF1-expressing macrophage subpopulation in the development of hypertension-related pathological progression. Neonatal exposure to hyperoxia is common in generating oxidant stress, leading to cellular senescence, bronchopulmonary dysplasia, and thymic involution and atrophy (CPhyS) [129]. In preclinical models, the administration of mesenchymal stem cell (MSC)-derived exosomes shows effective alleviation of oxidative stress and improves bronchopulmonary features and functions by modulating lung macrophages. Little is known regarding the restorative capability of mesenchymal stem cell-derived exosomes for hyperoxia-mediated lung injury. In this study, three thymi derived from three groups of mice subject to normoxia, hyperoxia, and hyperoxia^+^MSC exosomes were isolated and dissociated for scRNA-seq. The experiment demonstrated that MSC exosomes administered during neonatal hyperoxia exposure significantly restore thymic medullary architecture and immune functions. The scRNA-seq data analysis indicated that hyperoxia^+^MSC exosomes induced a transcriptomic regulation with 379 upregulated genes in *Siglech*+ DCs, 557 upregulated and only 23 downregulated genes in *Xcr1*^+^ DCs, and 339 upregulated and only 6 downregulated genes in *Aire*^+^ mTECs. The thymic DCs have a peripheral origin and migrate to the thymus to promote thymocyte maturation by antigen presentation.

Calcium phosphate ultra-small nanoparticles (CaP NSNPs) have been demonstrated to be more effective in therapeutic applications to restore osteoporotic bones than traditional NPs (CPS) [209]. Some studies have focused on single-organ toxicity of CaP NSNPs; however, systemic biosafety of these novel NPs has not been thoroughly investigated. Whole blood of rats at day 0, 3, and 14 after receiving CaP NSPs or hydroxyapatite nanoparticles (HANPs) was collected for CyTOF immune profiling at the single-cell level. Cell clustering analysis unveiled cell populations based on markers, such as CD4^+^ T cells (CD45^+^ CD3^+^ CD4^+^), CD8^+^ T cells (CD45^+^ CD3^+^ CD8^+^), γδT cells (CD45^+^ CD3^+^ TCRgd^+^), B cells (CD45^+^ B220^+^), neutrophil (CD45^+^ CD11b^+^ Ly6g^+^), macrophage (CD45^+^ CD11b^+^ F4/80^+^), monocyte (CD45^+^ CD11b^+^ Ly6c^+^), DCs (CD45^+^ CD11b^+^ CD11c^+^), and NK cells (CD45^+^ NK1.1^+^). After 3-day exposure, innate immune cells (monocytes, macrophages, neutrophils, NK cells, and DCs) were increased in the CaP USNC and HANP groups relative to the control group, whereas the proportions of B cells or CD4^+^ and CD8^+^ T cells were higher in the CaP USNC group compared to the control group. The CaP USNC group displayed higher expression of CD80, CD115, CD25, CD69, and CTLA-4 genes than the HANP and CON groups. At day 14 post-treatment, innate immune cells were all decreased, but adaptive immune cells were expanded, suggesting the adaptive system was activated. The team uncovered that CaP USNCs triggered stronger immune responses in five local tissues, including cartilage and chondrocytes, while improving liver and kidney metabolism. Moreover, according to the immune response profiles, data demonstrate that CaP USNCs are as safe as Food and Drug Administration-approved CaP nanoparticles at post-treatment day 14. In a study of smoking effects on genome toxicity of PBMC using single cell gel electrophoresis (SCGE), 82 subjects were grouped into smokers and nonsmokers and three age groups: young adults, adults, and older adults (CPhyS) [104]. SCGE data found that genomic instability was correlated with smoking, age, incorrect diet patterns, long sitting hours, and previous exposure to radiation. Takayasu arteritis (TA) is a vasculitis that primarily affects the aorta and its main branches. Recent studies indicated that IL-6 promotes the differentiation of CD4^+^ T cells into Th17 cells that secrete IL-17, IL-21, and IL-22 and cause an autoimmune response. It is still not clear why TA is still progressing even under targeted drug treatments. In order to elucidate the molecular mechanisms underlying TA, single-cell analysis was applied to transcriptomic profiling of PBMCs from a TA group and a control group (CPhyS) [122]. A total of 29,918 qualified cells were included for data analysis, and unsupervised clustering identified 10 clusters, including 8 annotated cell types and 2 unknown clusters. The proportion of memory CD4^+^ T cells in the TA group was increased, but that of naïve CD4^+^ T cells, cytotoxic CD4^+^ T cells, CD4^+^ T cells, CD8^+^ T cells, and NK cells was decreased. Meanwhile, the TA group had a higher proportion of CD14^+^ monocytes and B cells but a lower proportion of CD16^+^ monocytes, megakaryocytes, dendritic cells, and plasmacytoid dendritic cells. In exploring DEGs, the team found that CD14^+^ and 16^+^ monocytes had higher expression of CD163, AREG, THBS1, and TXNIP. Gene ortholog analysis indicated that the receptor binding for advanced glycation end products (RAGE) pathway was activated in CD14^+^ monocytes and dendritic cells. Interleukin family genes were highly expressed in the TA group, including IL-6, IL-6STP1, IL-6ST, IL-15, IL-15RA, IL-18, IL-18RAP, and IL-18R1. IL-6 was dominantly present in B cells and CD16^+^ monocytes. The expression of genes in the TA group suggested that inflammation and oxidative stress may play a role in the TA disease. Severe burns are usually characterized by a feature of T cell immune suppression and cause multiple organ damage and failure (MOF), systemic inflammatory response syndrome (SIRS), or sepsis. COVID-19 infection sustained innate immune activation and an insufficient adaptive immune response with reduced dendritic cell number and compromised CD80 and CD86 co-stimulatory molecules. The pathological immune deprivation similarity suggests that different stressors may elicit a shared molecular response (APS/APhyS) [96]. ScRNA-seq of COVID PBMC showed 15 clusters of cells using a shared nearest neighbor (SNN) modularity optimization-based clustering algorithm. Remarkably, common CD86-centered immune network genes, namely stress response core (SRC) genes, were robustly expressed in both burns and COVID disorders and indicated better clinical prognosis. The genes, such as CD86, CD1C, and HLA-DPA1, which were essential to T cell proliferation and activation, were highly expressed in the SRC network.

## 7. The Reproductive System

Stress, including aging, has deleterious effects on the reproductive system, although the harmful consequences at the single-cell level are emerging [210,211]. The primordial follicular pool is responsible for female reproductivity and fertility, established in the embryonic stage and diminished by aging. Granulosa cells (GCs) are crucial for the maintenance of the follicular pool. In silico analysis of scRNA-seq of 4 young and 4 aged nonhuman primate ovaries and *t*-SNE plots showed 14 ovarian cell types, with cluster 3 corresponding to GCs based on four of the known specific GC markers: *AMH*, *WT1*, *INHA*, and *CYP19A1* (CPhyS) [101]. PCA and *t*-SNE uncovered 20 clusters with distinct transcriptomic profiles. The data indicated that upregulated MAPK1 serves as a protective factor against the aging phenotype in aged nonhuman primate and mouse models [101]. The study by Yang et al. (2022) investigated the anti-aging effects of Coenzyme Q10 (CoQ10) on oocytes (CPhyS) [171]. A total of 27 potential therapeutic targets were screened, with seven hub targets (PPARA, CAT, MAPK14, SQSTM1, HMOX1, GRB2, and GSR) identified. Functional and pathway enrichment analysis indicated that these 27 putative targets exert therapeutic effects on oocyte aging by regulating signaling pathways such as PPAR, TNF, apoptosis, necroptosis, prolactin, and MAPK signaling pathways. They are also involved in oxidation-reduction processes, mitochondrial function, enzyme binding, reactive oxygen species metabolic processes, and ATP binding. Additionally, five densely linked functional modules revealed that CoQ10 improves aging-related deterioration of oocyte quality through mechanisms closely related to antioxidant activity, mitochondrial function enhancement, autophagy, anti-apoptosis, and immune and endocrine system regulation. Molecular docking studies revealed that the seven hub targets have a good binding affinity towards CoQ10, and molecular dynamics simulations confirmed the stability of the interaction between the hub targets and the CoQ10 ligand. These findings suggest that CoQ10 targets several key proteins and pathways involved in oxidative stress and mitochondrial function, thereby enhancing oocyte health and potentially improving fertility outcomes. In a study to investigate the effects of maternal age on mature oocytes, a scRNA-Seq analysis identified 357 DEGs between mature oocytes from older and younger women (CPhyS) [212]. The genes with increased expression in aged oocytes were enriched in the signaling of transcriptional activation, oxidative stress, and immune function, while downregulated genes were involved in catalytic activity. *TOP2B* appeared crucial for oocyte quality and early embryo development by protein interaction network analysis and knockdown verification on younger mouse mature oocytes. The comparison of ovarian cells of 3- and 9-month-old mice indicated the decline of granulosa cells, primordial and tertiary follicles, but a more than 2-fold increase in immune cells, including B cells, conventional T cells, and innate-like T cells, in aged female ovaries (CPhyS) [86]. Another mouse aging model study showed that scRNA-seq analysis of oocytes isolated from 5-week-old and 32-week-old female KM mice allowed the evaluation of the aging impact on oocyte quality (CPhyS) [213]. The scRNA-seq data uncovered 624 DEGs between two age groups of mouse germinal vesicle (GV)-stage oocytes. In the aging GV oocytes, 449 DEGs were upregulated and 175 DEGs were downregulated [213]. Among the downregulated genes, oxidative phosphorylation and the ATP production pathway in mitochondrial function were significantly enriched in KEGG pathway enrichment analysis in GV oocytes of 32-week-old mice. These genes were the mitochondrial encoded NADH dehydrogenase (mt-Nd) specifically, including mt-Nd2, mt-Nd3, mt-Nd4, mt-Nd4L, and mt-Nd5. Additionally, the expression of endoplasmic reticulum stress-related genes, including *AdipoR2*, *IRAK-1*, *RCAN1*, and *Msrb1*, and anti-oxidation-related genes (*Erbb3*, *Rcan1*, *Gsto2*, and *Msrb1*) was also significantly decreased in the elderly mice. The impacts of aging on the male reproductive system have not been fully investigated [65]. A study focusing on testicular cells of a cohort of 35 males aged 21–69 uncovered two major aging changes: increased peritubular cell basement membrane thickness in 30 s, and testicular cell functional changes, altered steroid metabolism in Leydig cells, and immune responses in macrophages, in 50 s. Chronic psychological stress of audio shocks has been shown to affect male delayed spermatogenesis, sperm production, and quality (CPsyS) [95]. Global transcriptomics of single sperms indicated downregulation of anti-oxidation-related genes (*GPX1* and *STAR*) and upregulation of genes (*Fos* and *F3*) promoting reactive oxygen species in stressed rats.

Fertilized ova and embryos experience negative impacts from stress, too. In a previous study, Rodgers et al. found that males exposed to chronic stress produced offspring with a dampened HPA axis response and reprogramming of gene expression in the hypothalamic PVN, but the mechanistic causes remained unclear [214]. Later, they recapitulated significant paternal stress effects on reduced HPA axis responsivity through microinjection of nine microRNAs that were postulated to carry out the effects in sperms (CPsyS) [130]. Single-cell qRT-PCR using the BioMark HD System robustly validated the dysfunction of maternal gene mRNA being targeted by the paternal microRNAs. Moreover, they associated long-term reprogramming of the hypothalamic transcriptome with HPA axis dysfunction, revealing that a remarkable reduction in the expression of extracellular matrix and collagen gene sets leads to blood–brain barrier permeability changes. The implantation of embryos into the endometrial tissue poses nutritional stress to the embryos and causes failure of implantation. The embryonic response to nutritional stress is still largely unexplored [215].

Single-cell RNA-seq analysis was carried out on 19 samples at five early embryonic development stages (zygote, 2-cell, 4-cell, 8-cell, and morula stages) (APS). There were increased DEGs from zygote to 2-cell stage and from the 2-cell to 4-cell stage, with 52 and 6404 DEGs, respectively. A total of 900 DEGs were found between the 4-cell and 8-cell stages. PCA identified three groups of cells based on the DEGs mentioned above. Upon zygotic genomic activation (ZGA), transitioning from the 2-cell stage to the 4-cell stage, arginine metabolism-related genes are apparently increased. Removal of arginine from culture medium led to decreased ZGA marker genes and SIRT1 protein in four cell porcine embryos. When arginine was added, the glutathione, ATP levels, and lipid droplet contents were significantly increased, but reactive oxygen species (ROS) were decreased. The data indicate that arginine plays an important role in stress response during embryonic implantation. Embryonic stem cells (ESCs) arise at the blastocyst stage before uterus implantation and thereafter differentiate into multiple cell lineages during gastrulation. Upon implantation, embryos encounter stress, eliciting a stress response by diminishing ATP, ESC pluripotency, and cell growth. Meanwhile, ESCs overcome the stress and differentiate into extra-embryonic endoderm and its sub-lineages. The driving transcription of genes for the process is still elusive. In an in vitro culture stress study, scRNA-seq data depicted that stressed cells treated with a hyperosmotic agent (sorbitol) have slower proliferation, fewer genes expressed, but more Gene Ontology groups (CPhyS) [133]. UMAP analysis identified eight clusters, with the majority of unstressed cells in clusters 0-1, whereas stressed cells were in clusters 2-7. High expression of markers suggested that cluster 7 includes early extra-embryonic endoderm sub-lineages (primitive endoderm and parietal endoderm), while cluster 6 is a later sub-lineage (visceral endoderm). A stress-induced cluster with transient intermediate cells expressing higher Stat3, Klf4, and Tbx3 (LIF receptor downstream genes) lies between the naïve pluripotency and primitive endoderm clusters. Sorbitol, like retinoic acid, also inhibits pluripotency and promotes lineage imbalance [133]. Preeclampsia is a complex syndrome characterized by high blood pressure and proteinuria occurring post-20th week of pregnancy. Abnormal placental imprinting and potential regulatory genes, including *GATA3* and *DLX5*, were found to play a role in preeclampsia (CPhyS) [183]. Depleted imprinting of upregulated *DLX5* was associated with classic preeclampsia markers in 69% of preeclamptic placentas. In in vitro trophoblasts, high expression of *DLX5* induced decreased proliferation, elevated metabolism, and endoplasmic reticulum stress-response activation. Enriched pathway analysis revealed that *DLX5* affected the signaling pathways in deregulated axon guidance, interleukin-8, and neuregulin receptor signaling; thyroid hormone receptor/retinoid X receptor, retinoic acid receptor, and planar cell polarity pathway; and antigen presentation pathway, unfolded protein response, and nuclear factor, and erythroid 2 like 2-mediated oxidative stress responses. Upregulated *DLX5* led to changes in ER stress response genes, *INSIG1*, *SREBF1*, *HSP90B1*, *ATF6*, *MB- TPS1*, *PPP1R15A*, *XBP1*, and *HSPA2*. The expression of *DLX5* in preimplantation in humans but not in mice supports the human-specific features of preeclampsia.

Occludins (ocln) and OZ proteins belong to tight junction components and are expressed in mammary gland development, but their precise roles in the process are not known yet. A previous study showed that mice with loss of ocln failed to nurse their young for uncertain reasons. Zhou et al. explored the single-cell transcriptomics of 910 ocln null and 1425 control luminal cells of normal mice and Ocln^−/−^ counterparts [196,197] using SMART-seq2. They identified milk-producing cells (MPCs) that still lack identifiable markers, and the mutant MPCs had 82 mostly upregulated and downregulated genes, with 14 upregulated genes involved in the endoplasmic reticulum (ER) stress and unfolded protein response (UPR) pathways (CPhyS) [216]. At the same time, 10 belonged to the apoptotic signaling pathway. MPCs were subject to ER stress as protein production increased exponentially during late pregnancy and lactation. Compared to normal MPCs, Ocln^−/−^ counterparts experienced high ER stress that led to increased apoptosis and acute shutdown of protein expression, eventually causing lactation failure in the female mice. The increased ER stress was caused by defective exocytosis of milk proteins in Ocln null cells. To confirm the function in the secretion of milk proteins, Ocln was seen to be colocalized on secretory vesicles and bound to SNARE proteins. Chemotherapy resistance is the major contributor to cancer mortality and is not well characterized yet. To explore chemotherapy resistance progression, high-grade serous ovarian cancer tissue samples from 11 patients were collected before and after chemotherapy, and transcriptomics were analyzed at single-cell resolution. With the aid of a novel analysis, PRIMUS, the team led by Dr. Vähärautio, found that chemotherapy promoted a constant increase in stress-associated cell state that was validated by RNA in situ hybridization and bulk RNA sequencing (CPhyS) [217]. From 93,650 cells, PRIMUS analysis identified 12 cell clusters: 3 patient-specific and 9 shared across multiple patients. Enriched pathway analysis discovered 10 pathways of diverse biological processes. The stress-associated cell profiles were present before chemotherapy, clonally enriched through the chemotherapeutic application, and predicted lower progression-free survival. An inflammatory cancer-associated fibroblast subtype coexists in tumors and implicates that chemotherapy-driven stress response in both cancer cells and stroma induces a paracrine feed-forward loop.

## 8. The Gastrointestinal (Digestive) System

Stress is closely associated with functional impairment of the gastrointestinal system [218,219]. Non-infection inflammation induced by gastrointestinal graft-versus-host disease targets intestinal stem cells (ISC), leading to their loss and subsequent morbidity and mortality in allogeneic hemopoietic stem cell transplantation (CPhyS) [126]. Previous studies indicated that cellular metabolism is central to the regulation of ISC survival and regeneration through epigenetic reprogramming via oxidative phosphorylation metabolites, e.g., alpha-ketoglutarate (alpha-KG) and succinate. In order to investigate the effects of graft-versus-host disease on ISCs, allogenetic and syngenetic bone marrow transplantations were performed in BALB/c → C57BL/6 and C57BL/6 → C57BL/6 groups, respectively. The scRNA-seq of Lgr5^+^ISCs demonstrates that the top differentially increased genes are *ido1*, *iigp1*, *cxcl9*, and *gbp2* and decreased genes are *fth1*, *chchd2*, *slc25a5*, *gpx4*, and *kif5b*. They reveal that metabolic adaptation in Lgr5^+^ISCs is associated with a reduction in succinate dehydrogenase complex flavoprotein subunit A (SDHA), resulting in the accumulation of succinate [126]. Previous inflammation insult experience shapes epigenomic imprint in ISCs, mediating subsequent cellular dysfunctions and vulnerability. Weaning poses stress on intestinal epithelial cells, inducing functional changes and an inflammatory response. In order to elucidate the changes afflicted by weaning, comprehensive transcriptional profiling [196,197] of 149 ileal cells from normal piglets and post-weaned counterparts derived 31 cell subtypes, including epithelial (enterocytes and epithelial secretory cells), stromal, and immune cell lineages (APhyS) [142]. Clustering analysis subdivided secretory cells into Paneth, goblet, enteroendocrine, *BEST4*/*OTOP1* cells, and enterocytes. *BEST4*/*OTOP1* cells display potential functions of electrolyte balance, sour taste, guanylate cyclase activator activity, and pH sensing in the release of sequestered calcium ions into cytosol, lipoprotein transporter activity, and the regulation of exocytosis. Unsupervised pseudotime trajectory analysis depicted that enterocytes initiate the lineage path, and four secretory cells are distributed along the terminal end. The ileal gene signatures of piglets are consistent with human and marine counterparts. *t*-SNE analysis identified B cell counts of the ASC2 subcluster, which is enriched in pathways involved in the intestinal immune network for IgA production, antigen processing, and presentation, and is different between the suckling and post-weaned groups. Weaning induced widespread mitochondrial damage in ileal epithelial cells with overexpression of cytochrome family genes (COX2, COX3, and CYTB) and anti-apoptotic and pro-apoptotic genes (*BCL2*, *BAX*, BH3-only family genes, *BIK*, *BIM*, and *BAK1*). In the weaning group, the increased Th17 subcluster showed differentially expresses genes enriched in the TNF signaling pathway, intestinal inflammation, and cytokine–receptor interactions. Cell–cell interaction analysis indicated that the Th17 subcluster is activated by local cytokine milieu from dendritic cells, macrophages, and epithelial cells [142].

Islets of the pancreas and liver are the two digestive organ tissues targeted by stress signaling. Sensing stress triggers a stress response in pancreatic β cells that undergo a transcriptomic shift and dedifferentiation into embryonic and neonatal β cell-like phenotypes, leading to malfunction of insulin secretion. The reversal of dedifferentiation of defective B cells by noncanonical Wnt signaling has shown therapeutic effects on restoring insulin endocrine function. Unsupervised graph-based clustering of 18,716 single endocrine cell transcriptomes revealed four major cell clusters, including α-, β-, δ-, and PP-cells (CPhyS) [135]. The heterogeneous β-cells were further classified into five clusters, with cluster 2 showing differential expression of CD81. High CD81 expression is associated with age, stress-mediated dedifferentiation, and ER stress of β-cells and decreased insulin secretion. Diabetic disease and in vitro stress studies identified CD81 as a novel surface marker that labels immature, stressed, and dedifferentiated β-cells in the adult mouse and human islets [135]. This novel surface marker will allow us to better study β-cell heterogeneity in healthy subjects and diabetes progression. Reduction of insulin secretion caused by dysfunction of pancreatic β-cells and/or decrease in β-cell mass is the primary inducer for Type 2 diabetes. The partial pancreatectomy model has been used to study the adult β-cell self-replication mechanisms to enlarge the β-cell mass and improve insulin secretion. However, β-cell self-replication gene networks were not thoroughly recapitulated by a microarray analysis study (APS) [144]. ScRNA-seq of islet cells from partial pancreatectomy and control mice and UMAP detected six subpopulations: clusters 1-4 (*Ins1*-expressing b-cells), cluster 5 (*Ppy*- or *Sst*-expressing PP- and δ-cells), and cluster 6 (*Gcg*-expressing a-cells). High expression of cell-cycle-related genes, *Mki67* (Ki67) and *Pcna*, in cluster 4 indicates replication activity. Pseudotime course analysis recapitulated the β-cell replicating state with observed transitional switching expression of cyclins from G0/G1, S to G2/M phase along the trajectory. The decreased expression of β-cell function genes such as *Ins1*, *Ins2*, *Pdx1*, *Nkx6.1*, *Neu-rod1*, *Ucn3*, and *Esrrg* in cluster 4 cells indicated a transitional progress of dedifferentiation. The beta-cell regeneration has been intensively studied, but the response of delta-cells after pancreatectomy is relatively unexplored. Pancreatic delta-cells exhibited a 1.5- to 2-fold increase in replication 4 weeks post-pancreatectomy (APS) [172]. ScRNA-seq identified four endocrine cell populations (alpha, beta, gamma, and delta), leukocytes, endothelial, and mesenchymal cells. Alpha cells were decreased while gamma cells were enriched. Beta cells are heterogeneous and were grouped into three subpopulations featuring stress-associated genes (*Fkbp11*, *Dapl1*, *Creld2*, *Sdf2l1*, *Pdia4* and *Derl3*), cell-cycle-associated transcripts (*Top2a*, *Ccna2*, *Ube2c*, *Cenpf* and *Nusap1*), and a stable transcript signature like the sham group. A unique subpopulation was composed of delta-interacting beta-cells expressing dominant beta-cell transcriptomes and partial delta-cell transcriptomes, indicating close physical interactions between the two subpopulations.

The study by Yang et al. (2023) investigates the potential benefits of N-acetylcysteine (NAC) in treating nonalcoholic fatty liver disease (NAFLD) (CPhyS) [170]. The results showed that NAC treatment significantly improved systemic and hepatic lipid metabolism (*p* < 0.01), reduced inflammation-related liver injury (*p* < 0.01), alleviated glucose intolerance (*p* < 0.05), and decreased hepatic steatosis (*p* < 0.01) by restoring hepatic glutathione (GSH) (*p* < 0.05) and GSH reductase (*p* < 0.05) levels compared to controls in NAFLD-induced animals. Consistently, in bulk, single-cell, and spatial transcriptomics data, the target pathways of NAC were strongly associated with NAFLD development in mice and patients. scRNA-seq analysis of mouse hepatocytes after 24-week treatment of Western diets showed that FFA uptake (Cd36), TG synthesis (Acsl1, Dgat1, and Dgat2), and GPx (Gpx1 and Gpx4) were increased. Additionally, the 10× Genomics Visium spatial transcriptomes elucidated that NAFLD patients have the upregulated expression of FFA uptake (CD36) and TG synthesis (ASCL1, MOGAT2, DGAT1, and DGAT2), GPx (GPX1 and GPX4), and fibrosis (COL1A1) and downregulation of b-oxidation (PPARGC1A and CPT1A), GR (GSR), and insulin signaling (AKT1). Through a combination of transcriptomic analysis and meta-analysis of preclinical studies, the researchers found that NAC holds promise as a therapeutic agent for NAFLD and warrants further clinical trials. The effects of diets on pancreatic islets and the development of type 2 diabetes have not been well defined. Three months of high-fat, high-fructose (HFHF) diet treatment induced metabolic disorders and enlarged the islets in the pancreas, which were then decreased and became irregular after 18 months. The HFHF diet resulted in significantly higher fasting insulin, C-peptide, proinsulin, and intact proinsulin levels than those in matched control rats on normal diets [220]. From a total of 78,024 qualified cells of six rats of three groups (young, HFHF-old, and controlled old), 10 clusters of cells were identified using unsupervised clustering analysis. The identities of clusters were defined based on the markers *Cd3e* and *Cd8a* (T cells), *Ccl24* and *Cd83* (macrophage cells), *Cpe* and *Pnliprp1* (secretory cells), *Postn* and *Ccdc80* (fibroblast cells), *Irf8* and *Ly86* (B cells), *Aqp1* and *Flt1* (endothelial cells), *Acta2* and *Myh11* (vascular smooth muscle cells), and *Sox9* and *EpCAM* (ductal epithelial cells). The HFHF diet appeared to elevate oxidative stress and subsequent inflammatory factors according to increased plasmatic oxidative parameters and nucleic acid oxidation markers (8-oxo-Gsn and 8-oxo-dGsn). Marker transcripts allowed the team to sort out four endocrine cell groups in islets, alpha cells (*Gcg* and *Gc*), beta cells (*Ins1* and *Pcsk2*), polypeptide cells (*Ppy* and *Pyy*), and delta cells (*Sst*), and acinar cells that were all decreased significantly in the HFHF-old group. Gene Ontology Analysis demonstrated that an evident enrichment of inflammation and immune-related pathways was found in the HFHF-old group compared to the old group. Single-cell RNA sequencing also validates the different modifications of oxidoreductase transcription in islet subpopulations with aging and long-term HFHF diet.

## 9. The Circulatory (Cardiovascular) System

There is robust evidence for cardiovascular disease development and progression with psychological stress [221,222]. In addition to the HPA axis, the renin-angiotensin-aldosterone (RAA) system, an endocrine system, is also excited under stress to promote angiotensin II to regulate blood pressure and volume [223]. The administration of angiotensin II as a chronic stress model was used to study stress effects on heart tissues. Heart tissues were harvested for pathological assessment and single-nucleus RNA-seq from mice subject to 2 weeks of implanted injection of angiostatin II and sham animals (CPhyS) [111]. A variety of cell types were present in all heart tissues including fibroblasts (*Pdgfra*, *Col1a1*), pericytes (*Pdgfrb*, *Vtn*), smooth muscle cells (*Acta2*, *Myh11*), Schwann cells (*Plp1*, *Kcna1*), endothelial cells (*Pecam*, *Ly6c1*), macrophages (*Fcgr1*, *Csf1r*), and other immune cell populations (granulocytes, B cells, T cells, and natural killer cells). Further analysis identified two fibroblast subpopulations that dramatically increased in the stressed group as compared to the unstressed counterparts. The two subpopulations were referred to as fibroblast-*Clip* and fibroblast-*Thbs4*. The study also obtained the detailed transcriptomic networks for normal homeostasis and pathological hypertrophy. The data showed that cardiomyocytes are the principal producers of *Vegfa*, which is an important factor for endothelial and angiogenic growth. The study by Whitehead and Engler explored the regenerative interactions between cardiac cells and macrophages in the heart (CPhyS) [164]. The researchers used RNA sequencing (RNA-Seq) datasets to analyze the contributions of cardiac fibroblasts (CFs) and macrophages in the healing process of both regenerative (postnatal day 1) and nonregenerative (postnatal day 8+) hearts. They found that nonregenerative hearts exhibited increased extracellular matrix (ECM) production and inflammation, leading to profibrotic gene programs that hinder regeneration. The study suggests that the shift in macrophage ontogeny postnatally results in stress signaling that suppresses heart regeneration. Single-cell calcium tracing has identified that time-based measures of relaxation, including the T50 (*p* = 0.04) and time to maximal reuptake velocity (*p* = 0.02), were significantly prolonged in the 47 K mutTg mice, and AAV9 M7.8L therapy treatment yielded a complete recovery to normal values (T50, *p* = 0.03; time to maximal reuptake velocity, *p* = 0.002). The whole-transcriptome analyses further revealed no significant changes in argonaute (*AGO1*, *AGO2*) and endoribonuclease dicer (*DICER1*) transcripts, and endogenous microRNAs were preserved, suggesting that the RNAi pathway was not saturated (CPhyS) [178]. Data suggested that disturbed shear and pulsatile shear have different effects on endothelial cells of the aorta [61]. ScRNA-seq of the straight parts of the arterial tree and the inner curvature of the aortic arch and bifurcation revealed that disturbed shear promotes endothelial-to-mesenchymal transition, with upregulation of Enolase I (*Eno1*), leading to increased inflammation, hypoxia responses, TGF-β signaling, glycolysis, and fatty acid synthesis. Subject to hemodynamic stress, thoracic aorta cells, particularly aortic smooth muscle cells (SMCs), undertake adaptive remodeling to increase signal and gene expression to facilitate proliferation, extracellular matrix production and wound healing to increase aortic wall thickness to sustain abnormal pressure (CPS) [191]. To explore the cellular changes, single-cell RNA-seq and ATAC-seq were applied to interrogate the aortic SMC gene expression and epigenetic modifications in an angiostatin II-induced mouse model fed a high-fat diet. Sc-RNA-seq analysis revealed nine major cell clusters and an adaptive response in thoracic SMCs displaying enriched pathways with increased gene expression of wound healing, elastin and collagen production, proliferation, migration, cytoskeleton organization, cell–matrix focal adhesion, and PI3K-PKB/Akt (phosphoinositide-3-kinase-protein kinase B/Akt) and TGF-β (transforming growth factor beta) signaling [191]. ScATAC-seq analysis identified 3 SMC clusters from 13 major cell clusters with increased chromatin accessibility at regulatory regions of adaptive genes and revealed a mechanical sensor, YAP transcription cofactor, responsible for the expression of these genes (e.g., *Lox*, *Col5a2*, *Tgfb2*). Knockout of SMC-specific *Yap* in mice attenuated this adaptive response and resulted in aortic aneurysm and dissection (AAD) incidence. Due to the complexity, it is still difficult to predict atherosclerosis and its progression. Reevaluation of scRNA-seq data led to the finding of upregulated and downregulated differentially expressed genes related to ferroptosis, pyroptosis, and necroptosis gene sets in atherosclerosis. The results provided nine immune cell clusters and two novel predictors, ALOX5 and NCF2, that were highly expressed in monocytes and M1 and M2 polarized macrophages for atherosclerosis (CPhyS) [92].

The study by Xu, Jin et al. (2022) investigated the mechanisms behind heart failure in patients with hypoplastic left heart syndrome (HLHS) using induced pluripotent stem-cell-derived cardiomyocytes (iPSC-CM) (CPhyS) [167]. It was found that iPSC-CM from patients with HLHS who exhibit early heart failure demonstrated increased apoptosis, mitochondrial respiration defects, and redox stress due to abnormal mitochondrial permeability transition pore (mPTP) opening and a failed antioxidant response. Conversely, iPSC-CM from patients without early heart failure displayed normal mitochondrial respiration and an elevated antioxidant response. Single-cell transcriptomics further confirmed the association of early heart failure with mitochondrial dysfunction and endoplasmic reticulum (ER) stress. ScRNA-seq of 4430 iPSC-CM was obtained from group I (surviving transplant free) and group II (heart transplant at 7 months or deceased at 2 months) HLHS patients, and the control formed nine cell clusters. Group I was enriched in protein translation and cell cycle genes, whereas group II had high expression of hypoxia and mitochondria-related genes, including ATP synthesis and oxidative phosphorylation pathways. These findings suggest that oxidative stress, which is not compensated for, underlies the early stages of heart failure in HLHS cases. Notably, the study also identifies that sildenafil treatment to inhibit mPTP opening or TUDCA to suppress ER stress can rescue the observed mitochondrial respiration defects, oxidative stress, and apoptosis. This highlights the importance of maintaining mitochondrial health as a critical factor in managing congenital heart diseases. A study has shown that exercise can promote pulsatile shear stress in arterial circulation, exhibiting beneficial contributions to reducing cardiometabolic diseases (CPS) [52]. In the wheel-running animal model, exercise upregulates endothelial stearoyl–CoA desaturase 1 (SCD1) which ameliorates NF-kB proinflammatory response by producing lipid metabolites, oleic and palmitoleic acids.

## 10. The Respiratory System

Rajan et al. studied molecular adaptation of osteosarcoma in response to novel microenvironmental stressors in lungs during metastasis [125]. In lung colonization, the study identified adaptive transcriptional changes, but tumor phenotypic diversity still remained despite clonal selection (CPhyS) [125]. The shifts in transcriptomes of tumor cells were related to several pathways of energy metabolism. Genes that were upregulated in tibia colonization were associated with glycolysis, hypoxia, MYC targets, fatty acid metabolism, and oxidative phosphorylation. On the other hand, gene sets upregulated in lung colonization were TNFα signaling via NFκB and EMT, whereas those significantly downregulated included MYC targets and MTORC1 signaling. Most interestingly, their data suggest that distinct tumor subpopulations collaborate and serve as a driver for the tumor’s persistent heterogeneity and lung tropism.

## 11. The Urinary (Excretory) System

Two ischemic acute kidney injury kidneys were subject to scRNA-seq, and the data were integrated with three normal kidney scRNA-seq datasets from the Gene Expression Omnibus for differentially expressed gene identification (APhyS) [140]. Fifteen cell clusters were revealed by UMPA plot, including mesangial cells, podocytes, endothelial cells, loop of Henle cells, proximal and distal tubule cells, principal and intercalated cells, from collecting ducts, macrophages, monocytes, dendritic cells, B cells, natural killer T cells, smooth muscle cells, and fibroblasts. DEGs were focused on tubular cells and endothelial cells, which are the major targets of ischemic injury. *RASSF4*, *EBAG9*, *IER3*, *SASH1*, *USP47*, and *SEPTIN7* were overexpressed in the kidney with ischemic injury and related to cell cycle control and apoptosis. They have not been reported in kidney injury. Pathway enrichment analysis identified endoplasmic reticulum stress (*PDIA6*, *ATF6*, *HSPA5*, and *DNAJC3*), regulation of apoptotic signaling pathway, retinoic acid-inducible gene I (RIG-I) signaling (*ANKRD17*, *BIRC3*, *PUM1*, and *LSM14A*), autophagy-related genes (*S100A11*, *CLDN1*, *TMBIM6*, *RB1CC1*, and *VMP1*) as well as antioxidants (*NQO1*, *GPX1*, *SOD2*, and *TXNRD1*). Ischemic kidney exhibited a proapoptotic and proinflammatory phenotype. The interplay between distinct kidney cell clusters in ischemic kidneys appeared to be most significant in macrophages with upregulated *SPP1*, *CCL3*, *CCL4*, *CCL4L2*, and *CXCL2* and monocytes with upregulated (CCL4, CCL4L2, CXCL14, CXCL12, and CXCL3) to interact with other kidney cell populations. Through in silico analysis of scRNA-seq data from renal transplantation patients (*n* = 3) with antibody-mediated rejection and acute kidney injury patients (control, *n* = 2), totaling 81, 139 cells were grouped into 21 clusters including 11 cell types, endothelial cells, epithelial cells, fibroblasts, macrophages, loop of Henle, stromal cells, proximal tubule, principal cells, B cells, CD8^+^ T cells, and intercalated cells (CPhyS) [160]. Fibroblasts, epithelial cells, loop of Henle, macrophages, and stromal cells were further classified into 8, 10, 6, 10, and 9 subpopulations, respectively. The dominant subpopulations in antibody-mediated rejection patients were Fibroblasts_PLVAP, Fibroblasts_CXCL9, Ep_S100A1, Ep_EGR1 LOH_ CLCNKA, LOH_JUN, Mac_FCN1, Stromal_ PDZK1IP1, and Stromal_MFAP5 cell subpopulations, compared to non-rejection cause patients. Previous evidence showed that diabetic nephropathy (DN) and metabolic syndrome (MetS) have a strong and complex relationship, but a causal relationship has not yet been proven (CPhyS) [186]. Discovery of biomarkers for DN and MetS would be beneficial in clarifying their interactions during disease progression. The DGEs found to be associated with both diseases were 86 upregulated genes and 22 downregulated genes that are enriched in immune system function, cellular function, and regulation of biological process. Further Cox regression analysis of the LASSO-selected data reduced the set to seven genes: *D52*, *CSGALNACT1*, *CX3CR1*, *SYK*, *TUBA1B*, *PAK2*, and *PLEKHA1*. Among the seven associated biomarker genes, the function of PLEKHA1 on oxidative phosphorylation (OXPHOS) in DN and MetS was validated using single-cell analysis [186]. *PLEKHA1* could initiate DN through the activation of B cells, proximal tubular cells, distal tubular cells, macrophages, and endothelial cells and subsequently induce OXPHOS monocytes in the kidney. The study by Wu et al. (2019) highlights the advantages of single-nucleus RNA sequencing (snRNA-seq) over single-cell RNA sequencing (scRNA-seq) in analyzing adult kidney tissue (CPhyS) [158]. The researchers found that snRNA-seq provides comparable gene detection to scRNA-seq while offering several benefits, such as reduced dissociation bias, compatibility with frozen samples, and elimination of dissociation-induced transcriptional stress responses. Additionally, snRNA-seq performed effectively on inflamed fibrotic kidney tissue, revealing rare cell types and novel cell states associated with fibrosis. From 11,391 transcriptomes, scDrop-seq generated exonic reads in the majority of mapped reads, while single-nucleus DropSeq (snDropSeq), DroNc-seq, and sn10× produced mapped reads that are intronic and not contaminated with mitochondrial transcripts. Combined datasets revealed 13 clusters of cells with podocytes, endothelial cells and mesangium, nine tubule clusters, and one macrophage cluster. Three snRNA-seq platforms outperformed scDrop-seq with higher sensitivity in detecting podocytes, endothelial cells, and intercalated cells and no detected stress response cluster. To validate the snRNA-seq protocol, transcriptomes of 6147 single nuclei from fibrotic and inflammatory kidney cells were sequenced, resulting in 17 cell clusters by *t*-SNE analysis. Five unique cell clusters were discovered with specific expression signatures, including proliferating proximal tubule cells (*Havcr1* and *Vcam*), dedifferentiated proximal tubule cluster (*Vcam1*, inflammatory *Ccl2*, *Il34*, *Cxcl1*, and *Cxcl2*), rare juxtaglomerular apparatus cells (*Ren1*), and two activated fibroblast subpopulations expressing either mannose receptor 2 or tenascin C and promoting fibrosis.

In order to investigate the therapeutic integration of mesenchymal stem cells (MSCs) in bladder vasculature in an interstitial cystitis/bladder pain syndrome (IC/BPS) mouse model, two-photon imaging analysis and single-cell microarray transcriptomic profiles were employed to monitor the process and dissect the molecular mechanisms (APhyS) [181]. The research group compared the single-cell transcriptomes of engrafted MSCs in IS/BPS mice and control cultured cells. MetaCore analysis indicated that MSCs in IS/BPS mice displayed two subpopulations with higher heterogeneity and expression of genes involved in the extracellular matrix, cell adhesion, and cytoskeleton, as well as YAP/TAZ and EGFR signaling pathways, compared to the control cultured cells. *Fos*, a collagen Type IV chain (*COL4A1*, *COL4A2*, and *COL4A5*), and *CDK1* were upregulated in engrafted MSCs for migratory, anti-inflammatory, and replicative functions. These genes’ upregulation was further confirmed by qRT-PCR and immunofluorescent imaging.

## 12. The Musculoskeletal System

Acute compartment syndrome (ACS) is commonly caused by tibiofibular fractures with chronic high pressure surrounding the deep fascia and inflammation. The transcriptomic and functional pathway changes in the deep fascia are potentially contributory to ACS. Sc-RNA-seq of deep fascia from three tibiofibular fracture patients and three control patients with osteosarcoma thigh amputation uncovered the stress-induced transcriptional landscapes of fibroblasts and immune cells (APS) [155]. From a total of 53,116 cells in two groups, there were nine clusters identified, including B cells (*CD79*, *CD19*, and *MS4A1*), cycling cells (*TOP2A* and *MKI67*), endothelial cells (*VWF*, *PECAM1*, and *CD34*), fibroblasts (*COL1A1*, *COL1A2*, and *DCN*), mast cells (*KIT* and *TPSB2*), myeloid cells (*FCGR2A*, *CD33*, and *ITGAM*), T cells (*CD3G*, *CD3E*, and *CD3D*), smooth muscle cells (*CNN1*, *ACTA2*, and *TAGLN*), and other subtypes. T cells were further classified into eleven cell subtypes. The high stress group had higher proportions of CD4 TCM, GATA3^+^ CD4 TCM, GATA3^+^ CD8 TCM, GZMK^+^IFN^−^act CD4 TCM, and Treg cells, as well as lower proportions of cytotoxic CD8 T cells, GZMK^+^ CD8 Teff cells, and innate lymphoid cells, than the control group. Clustering analysis further subdivided 4 macrophage, 5 fibroblast, 2 monocyte, and 3 dendritic cell subpopulations that display heterogeneous transcriptional profiles of genes and enriched pathways that play a role in shaping ACS. The findings implicate the malfunctions of the deep fascia in ACS, suggesting potential intervention targets. ScRNA-seq analysis of WI-38 human diploid fibroblasts was utilized to study side-by-side the senescent phenotypes following exposure to different triggers (replicative senescence, etoposide, ionizing radiation) (A/CPhyS) [156]. In the second set of experiments, Wechter et al. analyzed the progression of the senescent phenotype over time in the etoposide model [156]. The first analysis indicated that those senescent cells following replicative senescence displayed wide heterogeneity, while cells rendered senescent by ionizing radiation or etoposide showed gene expression patterns comparable to one another. The team performed clustering analysis and found four major cell groups: group 1 (clusters 5, 0), group 2 (clusters 1,3), group 3 (2, 4, and 7), and group 4 (cluster 6). Group 1 includes proliferating cells with high expression of *CKS2*, *HMGB1/2*, *TOP2A*, *PCLAF*, *PTMA*, *CCNB1*, and *MKI67*, promoting cell cycle and proliferation. Group 2 is composed of cells with abundant transcripts of *CDKN1A* and *GDF15* for growth arrest and senescence-associated secretory phenotype genes (e.g., *COL1A1*, *MMP2*, and *CCL2*). The majority of group 3 cells are ionizing radiation- and etoposide-treated populations highly expressing *TPM2*, *TMSB4X*, *S100A11*, and *FTH1* genes, while group 4 has upregulated long non-encoded mRNA and noncoding RNAs (lncRNAs), such as *NEAT1*, *MALAT1*, *XIST*, and *MEG3*. In order to understand the dynamic progression of transcriptomics of cell senescence, WI-38 cells were exposed to 50 uM etoposides for 0, 1, 2, 4,7 and 10 days and subject to scRNA-seq and RNA velocity analysis. Clustering analysis of cells with sequenced transcripts delineated clusters 0-6 of populations 6, 1, and 4, with high expression of cell proliferation genes representing 80% cells at day 0 with no treatment, dropping to 30% at day 1 and further declining along the etoposide exposure. Cluster 0 grew from 15% at day 0 to 30% at day 1 and remained constant. Cluster 2 appeared to be a transitional population, with a spike at day 1 followed by a decline. Finally, clusters 3 and 5 were low at 1–3% at days 0–2 and expanded to 15–20% at day 4 and beyond. Cluster 3 was enriched in membrane and oxidative phosphorylation and senescence gene expression (*CCND1*, *CCND2*, *UCHL1*, and *CDKN2A*), whereas cluster 5 displayed decreased oxidative phosphorylation but increased GTPase activity. They appeared to be novel clusters of senescence cells during exposure to senescence inducers. In summary, single-cell transcriptomic analysis allowed the team to identify the specific populations and the dynamic transition states during senescence initiation and progression. Transcriptional profiles of 65,835 cells from five normal talus cartilages exhibited the atlas of chondrocytes and 16 cell clusters (0–15) (CPS) [159]. Based on gene signatures, clustering analysis identified two novel cell clusters, MirCs and SpCs, that have not been found in several osteoarthritis studies. The former cluster is characteristic of highly expressed *MT* and *HIF1* genes, whereas the latter group is composed of a low ratio of pct1/pct2 as an aging cell feature. Transcriptional profiles of proliferative chondrocytes (ProCs), homeostatic chondrocytes (HomCs), and regulatory chondrocytes (RegCs) are also different from those found in previous osteoarthritis studies and display novel functions in the regulation of cell death, a response to the steroid hormone and heparin binding and immune regulation. These novel chondrocyte clusters and novel functions of previously known chondrocytes are implicated in resistance to mechanical stress, repair of damage, and immune response of the talus.

Some data suggest that fibrodysplasia ossificans progressive (FOP) is induced by senescence-promoting activin A, IL6-STAT3, and other components of senescence-associated secretory phenotypes (SASPs) (e.g., PDGFs and MMPs) through steering reprogramming of myogenic cells into a chondrogenic cell fate. To understand the effective inducers of cell fate differentiation progression, single-cell RNA-sequencing (scRNA-seq) data of heterotopic ossification samples from mice subject to tenotomy and burn were utilized for in silico analysis (APS) [224]. Senescent cells in injured tendon tissues showed a stable increase from day 3 to day 21, with the highest level seen in this period. In a total of 38,689 cells from d0, d3, d7, d21, and d42 post-surgery, 16 cell clusters were identified, including macrophage cells, DC cells, granulocyte cells, NK cells, skeletal muscle cells, pericyte/smooth muscle cells, endothelial cells, tenocytes, fibroblasts, MSC, neuromuscular cells, nerve cells, skin fibroblasts, and a remaining small uncharacterized cluster. The heterogeneous expression profiles of Cyclin-Dependent Kinase Inhibitor 2A (Cdkn2a) and SASPs were not correlated with the senescence cell amount elevation trend. However, the increased senescent cells were significantly associated with ossification progression, such as ECM organization, cell adhesion, ossification, cartilage development, and so on, as shown in the GO (Gene Ontology) analysis. Senescence mechanisms for injury-related senescent fibroblasts (day 7 and 21) and age-related senescent fibroblasts (day 0 and 42) were apparently different, as depicted in different trajectories. The injury-related senescent fibroblasts demonstrate pathway enrichment in ossification, ECM remodeling, protein processing in PI3K-Akt signaling, MAPK signaling, focal adhesion, etc. Psychological stress suppresses skin response to Staphylococcus aureus skin infection by adipogenesis of skin fibroblasts through TGFβ and suppression of the antimicrobial peptide cathelicidin (*Camp*) [56]. Adrenergic inhibition, inhibitors of TGFβ signaling, and deletion of TGFβ receptors on fibroblasts restore the expression of *Camp* and skin immune defense.

Puram et al. mapped ~6000 tumor cell atlases under ecosystem stress from 18 head and neck squamous carcinoma cancer patients and five lymph node metastasis patients [119]. Copy number variation and EpCAM expression allowed them to distinguish the malignant (2215) and normal cells (3363), stromal cells, and immune cells. Among malignant cells, the expression pattern showed intratumoral and between-tumor heterogeneity in cell cycle, stress, epithelial differentiation, hypoxia, and partial epithelial-to-mesenchymal transition. The cells expressing a partial epithelial-to-mesenchymal transition pattern were localized to the edge of the tumor next to fibroblasts.

## 13. Discussion

In this review of 80 stress studies utilizing single-cell omics analysis, we identify (1) six classes of stressors, (2) single-cell omics analysis technologies and bioinformatics, and (3) a single-cell atlas of stress in different organs. Based on the duration and sources of stress, we classified stressors into acute physical, acute physiological, acute psychological, chronic physical, chronic physiological, and chronic psychological categories. Stress impacts single cells of the human body systems through molecular and cellular response, leading to pathophysiological contributions to physiological (e.g., hypoplastic left heart syndrome (HLHS)) and mental (e.g., major depressive disorder (MDD)) stress illness [54,167]. Additionally, cellular stress plays an influential role in entailing both physical and mental stress on the patients, especially in neurodegenerative, inflammatory, or autoinflammatory pathologies like PTSD and Parkinson’s Disease (PD) [25,33]. Understanding how stress underlies the progression and mechanisms of these pathological conditions at the single-cell level will allow us to effectively manage stress and design intervention strategies directed to prevent the escalation of maladaptive stress syndrome and improve human health and life quality. Previous efforts in pursuing molecular mechanisms of stress mainly relied on bulk studies of mixed cells, with oversight of cellular heterogeneity that limited progress toward understanding stress as an adaptive mechanism. The stress response is an adaptive mechanism by which the body attempts to cope with changes in the internal or external environment induced by stressors [18]. These stressors can arise from various physical and psychological stimuli that then contribute to the ongoing development of an adaptive response [18,225]. The form of these adaptive responses and whether they are proadaptive (beneficial to the patient) or maladaptive (harmful to the patient) are dependent on a litany of variables, including but not limited to stressor duration, perceived intensity, repetitiveness, and associated trauma [18,19,225]. Maladaptive stress responses often contribute to the development of neuropsychiatric conditions in conjunction with the longitudinal effects of the stressors [18,225]. These maladaptive stress responses can then cause significant damage identifiable at the cellular level [18,19].

Based on the criteria set in the methods, we reviewed 80 research articles on stress studies on human organ systems using single-cell analysis. The summary of these research papers is shown in Table 1. The organ systems studied comprise the nervous/endocrine, the immune, the cardiovascular, the urinary, the digestive, the respiratory, the reproductive, and the musculoskeletal systems, except the integumentary system. Almost half of the studies are on the nervous/endocrine and immune systems, reflecting the research preference and high interest in these two systems and their significant importance in the field. Depending on the abundance of cell sources and single-cell analysis throughput, there were as few as six oocytes and as many as ~415K cells processed for transcriptomic profiling [58,213]. These papers provide an in-depth single-cell omics landscape of multiple organ systems in response to variant stressors that are either acute or mostly chronic. The clinicopathological significance of chronic stress is apparent and reflected in its dominant proportion of the reviewed papers. The stressors involved have physical, physiological, or psychological origins, e.g., electric foot shock [69], pancreatomy [172], weaning [142], MDD, PTSD [58], etc.

Single-cell technologies and bioinformatics methods have experienced explosive growth, as shown in Table 1, and each method has its strengths and limitations, so benchmark comparisons have been extensively explored [226]. Those in-depth comparative performance studies on single-cell omics analysis approaches are available for consultation. Single-cell RNA-seq technologies vary substantially in mRNA capture efficiency, scalability, bias, cost, and optimal tissue applications [227]. In addition to technology platform variations, many technical and biological factors can introduce batch effects into omics data integration and skew the subsequent downstream analyses, including sequencing depth, read length, sample acquisition and handling, sample composition, and technical repeat. Batch correction algorithms have been developed for batch effect removal during data integration, and benchmark comparisons have shown that some methods outperform well, such as Harmony, scVI and scANVI [228,229]. By interrogating cellular dynamic processes and developmental relationships among single cells, the evaluation of 45 trajectory inference (pseudotime analysis) methods led to the development of guidelines for best practices in method selection for datasets [230]. The methods for single-cell multi-omics integration were also subject to systematic analysis [231].

The most used single-cell analysis technology among the reviewed studies is scRNA-seq, indicating that the application of other single-cell omics technologies, such as proteomics, epigenomics, metabolomics, and spatial transcriptomics, to study stress may deserve more attention in the field. Spatial transcriptomics not only investigates the transcriptomics at a nearly single-cell level but also conserves a spatial map of cells across the tissues [141]. Sc-proteomics can survey ~48 protein markers and their functions in cells [232,233] and is currently expanding into two-dimensional (spatial) proteomics to decipher protein distribution and localization within subcellular components [234]. Multi-omics to explore stress impacts, providing multiple layers of markers and cell-to-cell interactions, is still rarely utilized and urgently needed in the stress research field [121,235].

Physical stress is both inducive and derivative of multiple conditions and biological states (Table 1). These states vary wildly, ranging from underlying chronic high blood pressure to pancreatectomy [92,217]. Nevertheless, physical stress is often understood as exerting biomechanical stressors or otherwise involving pathways associated with extrinsic physical stimuli, such as in hypoxic stress and body-weight loading stress [119,236]. Physiological stress differs from physical stress; it is induced by chemicals or diseases [70,108]. Psychological stress typically affects cells via the psychoneuroimmunological pathways that can modulate various immune and inflammatory responses [19]. The immunoaffective stress responses may range from direct effects on cellular regulation and expansion to secreted cytokines that can indirectly dictate cellular function [19]. MDD and generalized anxiety disorder (GAD) are hallmark disorders in terms of these immunoaffective mental stress conditions [58,69,87]. Furthermore, multiple conditions are coexistent with both physical and mental stress or linked with sequential orders. Of these conditions, many are neurological in nature or are otherwise associated with the functioning of the nervous system. This form of stress is referred to as hybrid (physical or physiological/psychological) stress [69]. The pathophysiology of neurodegenerative conditions such as PD and AD illustrates that direct stress on neurons and other neuronal cells negatively affects myelination [70,110,121]. These hybridized stress conditions utilize the psychoneuroimmunological pathway that is also seen in mental stress, which can further add to the inflammatory damage seen in these conditions [19]. Of the three types of stress above, both physical and mental stress can be categorized as either acute or chronic. In a neuropsychiatric context, the most common differentiation between acute and chronic stress lies in the timeframe in which the stress is experienced [225]. When examining longitudinal stress periods, further differentiation is needed between repetitive stress (i.e., repeated acute stress) and prolonged stress (i.e., chronic stress) [18].

scRNA-seq has shown the differential landscape of stress transcriptomes in individual organ systems. The genes and pathways in the nervous system are particularly notable. The brain is the pivotal organ in processing stressors and executing the stress response, particularly the PVN, that mediates the initial stress response, CRH, and ACTH secretion. A single-cell transcriptomic atlas reveals that the PVN comprises highly heterogeneous cell subpopulations with diverse transcriptomic patterns. An acute stress study narrowed down secretagogin^+^ cells that are the CRH secretory cells, using Ca^2+^ sensing of secretagogin to coordinate vesicular traffic systems and exocytosis to deliver the hormone [131]. In a chronic stress model of ELA, active CRF^+^ neurons in adults are glutamatergic, with upregulation of heat-shock protein (*Hspa8* and *Hsp90ab1*), synaptic vesicle content and transport (*Vamp2*), membrane trafficking (*Atp5a1*), neuronal structure (*Actg1*), and translation (*Eif1*) [137]. Habenula is a center connecting stress and mood, fear, memory, and emotion, consisting of 26 cell clusters, increased neuroactive ligand–receptor interactions, and synaptic signaling when inflicted with chronic psychological stress [54,176]. The upregulation of *Lrrtm3*, *Grin1*, and *Kcnc1* in LHb neurons projecting to VTA is closely associated with chronic depression. The hippocampus executes fear memory through interactions between neurons and glial cells that are composed of 7 non-neuronal, 8 neuronal cell subtypes, and others. Acute stress triggers *Ttr*, *Ptgds*, and LTP genes in CA1 neurons, whereas chronic stress excites neuroimmune responses and neurogenesis in the dorsal hippocampus of animals resilient to stress [136]. The hippocampus loses glial lineage cells with enriched expression of inflammation and oxidative stress genes, leading to neurodegeneration under diabetic stress [108]. The epicenter of fear and anxiety, the amygdala, initiates an anxious mood by activating oligodendrocytes through xanthine derived from CD4^+^ T cells with upregulated IRF1 [69].

The prefrontal cortex is also a target of harmful stress. The DEGs for PTSD and MDD were identified mainly in excitatory and inhibitory neurons of DLPFC [58]. High glucocorticoid (GC) sensitivity of the HPA axis and low GC signaling are associated with PTSD, but the opposite is found in MDD. The deregulated pathways are involved in macrophage differentiation, metabolism, and mitochondria biology, immune response, tumor differentiation, and multiple enzyme functions that are operated by upstream regulators, including LPS, MYC, TGFB1, E2, and DEX. MDD also causes significant inflammation by recruitment of activated proinflammatory microglia [87]. PTSD-like fear induces microglia inflammatory response by activation and increased abundance [93]. Microglia depletion reduces PTSD-like symptoms. In addition to deep-layer excitatory neurons, MDD also predominantly affects gene expression of fibroblast growth factor signaling, steroid hormone receptor cycling, immune function, and cytoskeletal regulation in oligodendrocyte progenitors [88]. Chronic physiological stress effects of PD on brain dopamine neurons appear to include senescence induction and metabolic degradation in iPSC-derived neuron models and exhibit progressive pathological transcriptomic patterns with higher endoplasmic reticulum stress and enriched HDAC4-regulatory signaling [70,90,165]. Oxidative stress can be managed using FDA-approved felodipine, which has clinical potential for PD-related treatment.

The immune system is a major responder to stress, and several studies have revealed stress-stimulated global influence on hematopoietic stem cells and derived PBMCs. Burns and COVID-19 trigger a common CD86-led SRC gene signature in PBMCs [96]. The exposure to calcium phosphate nanoparticles induces increased subpopulations of innate immune cells at the immediate phase and then expands adaptive immune cells, e.g., CD8^+^ T cells, with higher expression of CD80 and CD25 [209]. Arteritis increases the proportion of CD^+^4 T cells and CD14^+^ monocytes with higher IL-6, IL-15 and IL18, but lowers the percentages of CD8^+^ T cells and NK cells and CD16^+^ monocytes [122]. Aging causes damage to HSCs in the bone marrow [153,157]. Aged mice have expanded HSC populations and a declining proportion of multipotent progenitor cells. Loss of *Aldh2* and *Fancd2* genes facilitates aging of HSCs by raising p53 pathway and aging signature genes. The response of HSCs to various stress sources is highly heterogeneous at the cellular level through epigenetic memory and regulation [179,194].

Aging stress slowly deteriorates granulosa cells (GCs) that maintain the integrity of follicular eggs and female fertility in the reproductive system by prohibiting MAPK1signaling [101]. The age-related degradation of oocytes could be prevented by CoQ10 treatment, which regulates PPAR, TNF, apoptosis, necroptosis, prolactin, and MAPK signaling pathways [171]. Weaning is a dietary change and results in stress in the gastrointestinal system of babies by regulating the immune network and coordinating IgA production and antigen presentation [142]. Overexpression of cytochrome family genes, increased Th17 T cells, and an enriched TNF pathway indicate widespread intestinal inflammation following the abrupt cessation of milk intake. In the cardiovascular system, chronic psychological stress prompts hypertrophic heart disease with a significant increase in two types of fibroblasts displaying marked expression of *Clip* and *Thbs4* [111]. In a hemodynamic stress model, aortic SMCs adopt adaptive strategies, increasing collagen, focal adhesions, and PI3K-PKB/Akt gene expression by upregulating chromatin accessibility via the Yap pathway [191].

As the single-cell omics atlas brings new insights into stress biology, there has been a dramatic increase in newly discovered cell types, DEGs, and enriched functional pathways involved in stress. Common stress-enriched pathways include synaptic and cellular transport, proinflammatory responses, oxidative stress, and other pathways. A crucial future research goal will be to integrate new cell types, DEGs, and enriched functional pathways across organs to uncover common stress mechanisms. The integration of single-cell omics data could be substantially improved by removing batch effects due to different single-cell technology platforms, tissue handling, sample sizes, sequencing depth, and bioinformatics methods. The integrative comparison will also lead to the identification of organ-specific orchestrating genes and stress principles. Those common and organ-specific DEGs and enriched pathways have great potential to serve as stress biomarkers for stress monitoring and therapeutic targets. They can also guide longitudinal studies, which have rarely been explored due to the high costs of single-cell omics, to define the hallmarks of stress during disease progression. With multi-omics approaches, the study of individual heterogeneity in stress responses would greatly advance precision medicine.

There are some limitations to this review, including its failure to cover single-cell omics across all organ systems and stress studies related to plants and microorganisms. Local impact of molecular and cellular stress is also beyond the scope of this review. Moreover, due to our single-cell focus, many findings relevant to the systemic and general stress responses are omitted without discussion. However, this review explores stress response and harmful consequences across a wide range of tissues and organs, using scRNA-seq and other omics. Acute and chronic stress generate distinct omics patterns, while physical, physiological, and psychological sources yield distinct omics outcomes. Although each study focuses on a specific organ, the single-cell stress omics atlas still exhibits substantial cellular heterogeneity and a global gene expression landscape across human organ systems. As foreseen, systematic stress studies with single-cell analysis and multi-omics across multi-organ systems exposed to multiple stressors will provide an unprecedented, comprehensive view of the complex stress syndrome and pave the way for novel therapeutic strategies.

## Figures and Tables

**Table 1:** Summary of stress-related single-cell analysis studies.

No	Author	Stress	Acute vs. Chronic	Physical, Physiological, or Psychological	Tissues	Single-Cell Technologies	Bioinformatics
1	Ba et al. [47]	Restraint	Chronic	Physical/ Psychological	The lacrimal gland	ScRNA-seq 10× Genomics Chromium	Cell Ranger v7.1.0 for demultiplexing, alignment, and UMI quantification, and conversion into Seurat objects (Seurat R v4.3.0.1) in [48]; Harmony v1.0 for batch effect correction [49]; RunPCA for dimensionality reduction; visualization with t-distributed Stochastic Neighbor Embedding (*t*-SNE)
2	Cathomas et al. [50]	Chronic social defeat stress	Chronic	Physical/ Psychological	Monocytes and myeloid cells in the brain	SMART-seq2 scRNA-seq	STAR v2.5 [51]; Seurat R v3.1.5
3	Cavallero et al. [52]	Pulsatile shear stress	Chronic	Physical	Aortas	ScRNA-seq 10× Genomics Chromium	Cell Ranger; Scanpy package in Python, Louvain clustering algorithm for cell type clustering [53]; PCA; UMAP; the heatmap. 2 in the “gplots” package of R
4	Cerniauskas et al. [54]	Chronic mild stress (CMS)	Chronic	Physical/ Psychological	Lateral habenula (LHb) neurons	ScRNA-seq; Clontech’s SMARTer Ultra Low RNA Input v4 or SMART-Seq HT kit	STAR; quality control and normalization using scran [55]
5	Chan et al. [56]	Restraint	Chronic	Physical/ Psychological	Skins infected with Staphylococcus aureus	ScRNA-seq 10× Genomics Chromium	Seurat in R; UMAP; *t*-SNE; R package CellChat for cell–cell communication analysis [57]
6	Chatzinakos et al. [58]	PTSD and MDD	Chronic	Psychological	The dorsolateral prefrontal cortex (DLPFC)	ScRNA-seq 10× Genomics Chromium	Cell Ranger v.3.1.0; Seurat v3.1.2; LIGER; *t*-SNE
7	Chawla et al. [59]	Major depressive disorder	Chronic	Psychological	DLPFC	SnATAC-seq	ArchR for single-cell chromatin accessibility analysis [60]; per-subject pseudo-bulked accessibility estimates
8	Chen et al. [61]	Disturbed blood flow	Chronic	Physical	Arterial tissues	ScRNA-seq 10× Genomics Chromium	Seurat v4.0.2; UMAP; SingleR for cell annotation [62]
9	Crinier et al. [63]	Acute myeloid leukemia	Chronic	Physiological	Natural Killer cells in bone marrow	ScRNA-seq 10× Genomics Chromium	Cell Ranger v3.0.0, v3.0.1; Seurat; Seurat’s FindClusters for cell clustering; PCA; SingleR for contamination; *t*-SNE; UMAP; FindAll-Markers for cluster markers; the pseudotime algorithm Monocle 3 DDRTree [64]
10	Cui et al. [65]	Aging	Chronic	Physiological	Testicular cells	ScRNA-seq 10× Genomics Chromium	Cell Ranger v.2.2.0; Seurat R; PCA; UMAP; CellChat; Mfuzz. analysis [66]
11	Daskalakis et al. [67]	PTSD & MDD	Chronic	Psychological	DLPFC	10× Genomics Chromium	Seurat and Gene set enrichment analysis (GSEA) [68]
12	Fan et al. [69]	Electronic foot shock (ES)	Chronic	Physical/ Psychological	Amygdala	ScRNA-seq 10× Genomics Chromium	*t*-SNE and unsupervised clusters
13	Fernandes et al. [70]	Rotenone and tunicamycin)	Acute	Physiological	iPSC-derived dopaminergic neurons	ScRNA-seq 10× Gnomics Chromium	Cell Rangers; Seurat; UMAP; Scanpy; Pathway enrichment analysis used Metascape [71]
14	Hikosaka et al. [72]	Maternal infection and repeated social defeat stress	Chronic	Physiological /Psychological	Brain	Single-cell spatial proteome	Cell Profiler v4.2.4 [73]; the histoCAT v1.76 [74], the Potential of Heat-diffusion Affinity-based Transition Embedding (PHATE) [75]; the Markov affinity-based graph imputation of cells (MAGIC) [76]
15	Hing et al. [77]	Forced interaction test	Chronic	Psychological	Prefrontal cortex	ScRNA-seq 10× Genomics Chromium	Cell Ranger; DESeq2 [78] or zero-inflated negative binomial model (ZINB)WaVE-DEseq2 workflow; clusterProfiler v4.2.2 [79] or EnrichR v3.2 [80]; MAGMA v 1.10 [81]
16	Hwang et al. [82]	PTSD	Chronic	Psychological	The dorsal lateral prefrontal cortex	10× Genomics Chromium scATAC, scRNA-seq, Xenium in situ transcriptomics	Cell Ranger v6.1.1; Pegasus v1.5.0 [83]; UMAP; PCA; Harmony; ArchR v1.0.2; Signac v1.11.0 [84]; MACS2 v2.2.9.1 [85]
17	Isola et al. [86]	Ovarian aging	Chronic	Physical/ Physiological	Ovarian tissue (Mice)	ScRNA-seq 10× Genomics Chromium	R Studio v.4.2.2; SoupX for cell calling; UMAP; Doublets removal by the DoubletFinder
18	Kokkosis et al. [87]	Major depressive disorder	Chronic	Psychological	Prefrontal cortex	10× Genomics Chromium (dataset from [88])	Seurat; UMAP; Monocle 3
19	Kos et al. [89]	Early life adversity (ELA)	Chronic/Acute	Physical/ Psychological	Brain tissue (Mice)	ScRNA-seq 10× Genomics Chromium	Cell Ranger v3.0.2; Scanpy v1.4.5; Python package diffxpy for gene expression analysis
20	Lang et al. [90]	Genetic stress	Chronic	Physiological	iPSC-derived dopamine neurons	SMART-seq2	PCA; Over- dispersion analysis (#179); GO enrichment analysis; Bayesian approach for disease trajectories
21	Lee et al. [91]	Early maternal separation and social isolation	Chronic	Psychological	Hippocampus (Mice)	ScRNA-seq 10× Genomics Chromium	Cell Ranger; Seurat R v4.0.5
22	Li et al. [92]	Atherosclerosis and oxidative stress	Chronic	Physiological	Atherosclerosis plagues of arteries	ScRNA-seq (Silico analysis)	Seurat tidyverse; Matrix R package
23	Li et al. [93]	Electric foot shocks	Chronic	Physical/Psychological	Prefrontal cortex (PFC), hippocampus (HP), and amygdala (AMY)	Single-cell mass cytometry	Visualization tool for statistical epistasis networks (viSNE) and heatmap analysis
24	Li et al. [94]	Artery occlusion and the chronic unpredictable mild stress (CUMS)	Chronic	Physical/ Psychological	Hippocampus (Mice)	ScRNA-seq 10× Genomics Chromium	Cell Ranger; Seurat v4.0.5
25	Li et al. [95]	Audio terrifying shocks	Chronic	Physical/ Psychological	Rat testicular tissues	Singleron Matrix scRNA-seq-GEXSCOPE Single-Cell RNA Library Kit	Celescope v1.8.1 for gene expression matrixes; featureCounts v1.6.2 software for UMI counts and gene counts; Seurat v4.0.4; UMAP; clusterProfiler v4.7.1.003; CellChat v1.6.1; R package biomaRt v2.48.3 for homology mapping
26	Liang et al. [96]	Burns and COVID-19	Acute	Physical/Physiological	COVID-19 peripheral blood monocytes	Whole blood scRNA-seq; Affymetrix U133 Plus 2.0 arrays for burnt skin; Agilent-039494 SurePrint G3 Human GE v2 8 × 60K Microarray	GEOquery [97]; weighted gene co-expression network analysis (WGCNA) v1.71 [98]; Differential Gene Correlation Analysis (DGCA) R package v1.0.2 [99]; Single Sample Gene Set Enrichment Analysis (ssGSEA) R package GSVA v 1.42.0; R package limma v 3.50.3; Serua; RcisTarget v1.14.0 for prediction of transcription factors of skyblue core genes [100]
27	Lin et al. [101]	Ovarian aging	Chronic	Physiological	Nonhuman primate ovaries	ScRNA-seq (In Silico analysis)	edgeR v3.6.2 for DEGs [102]; R package pROC v1.16.2 for the area under the curve (AUC) of the receiver operating characteristic curve (ROC); GSEA, Gene Ontology (GO), and Kyoto Encyclopedia of Genes and Genomes (KEGG) pathways for gene signatures discovery
28	Lin et al. [103]	Hypoxia	Chronic	Physiological	Mouse cerebral cortex	ScRNA-seq (In Silico analysis)	Seurat; PCA; UMAP; Monocle 3 v1.0 and Monocle 2 v2.4; velocyto.py v11.2 for cell velocity; SCENIC for cell regulatory network and clustering
29	Locken-Castilla et al. [104]	Smoking	Chronic	Physiological	Peripheral blood mononuclear cells (PBMC)	Single-cell gel electrophoresis	PCA
30	Luskin et al. [105]	Audio shocks, feeding, and odor stimulants	Acute	Physical	The locus coeruleus and perilocus coeruleus	10× Genomics scRNA-seq with spatial transcriptomics (Pixel-seq) [106]	Seurat v4.0; R v4.0.3; DoubletDecon v1.02 to remove suspected doublet cells [107]
31	Ma et al. [108]	Diabetes	Chronic	Physiological	Hippocampus (mouse)	ScRNA-seq 10× Genomics Chromium)	Cell Ranger v3.0; Seurat R v3.1.1; CellPhoneDB v2.0 for ligand receptor partners; PCA; *t*-SNE; Find Markers for DEGs identification
32	Ma et al. [109]	Major depressive disorder, chronic social defeat stress	Chroni	Physical/ Psychologica	Prefrontal Cortex	ScRNA-seq 10× Genomics Chromium	Cell Ranger v3.1.0; Seurat v4.0.5; UMAP; cell communication analysis based on CellphoneDB; WGCNA v.1.72.1 for cell-specific gene modules
33	Mathys et al. [110]	Alzheimer’s disease	Chronic	Physiological/Psychological	The prefrontal cortex (Brodmann area 10)	ScRNA-seq 10× Genomics Chromium	CellRanger v2.0.0; Scanpy; R packages scran v1.8.2 and scater v1.8.1 for single cell data manipulation and QC analysis; Seurat R v2.3.4; RUVseq v1.16.1 to remove unwanted variation from RNA data; metap v1.1 to compute aggregate *p*-values
34	McLellan et al. [111]	Angiotensin II	Chronic	Physiological	Hearts of mice	ScRNA-seq 10× Genomics Chromium	Cell Ranger v 2.1.1; Seurat v 2.3.4 and 3.0.2; PCA; *t*-SNE or fast interpolation-based *t*-SNE (FIt-SNE) [112]; MAST for differential expression [113]; the enrichDAVID in clusterProfiler R package; The Circlize R package for intercellular communication network [114]; Velocyto v0.17.17 for RNA velocity [115]; Slingshot v1.3.2 for cell group identification [116]
35	Mendiola et al. [117]	Inflammation of the epitope of myelin oligodendrocyte glycoprotein	Chronic	Physiological	Central nervous system, spinal cord, ROS+ innate immune cells	ScRNA-seq 10× Genomics Chromium	Cell Ranger v2.0.1; Seurat R
36	Millet et al. [118]	Alzheimer’s disease	Chronic	Physiologic /Psychological	Immune cells from the brains of AD mice	Bulk and 10× Genomics snRNA-seq/scATAC-seq, SMART-seq v4, and spaiial transcriptome Xenium	UMAP; chromVAR for chromatin accessibility analysis; SCENIC & SCENIC+; scVelo for CellRank; FlowJo for flow cytometry data analysis
37	Puram et al. [119]	Stress and hypoxia	Chronic	Physical	Tumor cells	SMART-seq2	RSEM for expression quantification [120]; *t*-SNE
38	Qian et al. [121,122]	Alzheimer’s disease (AD), Parkinson’s disease (PD), Huntington’s disease (HD), multiple sclerosis (MS), epilepsy (Epi), and chronic traumatic encephalopathy (CTE)	Chronic	Physiological/Psychological	Human cortex (CTX), hippocampus (HIP), white matter (WM), basal ganglia (BG), and astrocytes	10× Genomics Chromium scRNA-seq, snRNA-seq, and spatial transcriptomic	Seurat R v4.1.0; Harmony; NNLM R package v0.4.4; the gene set variation analysis (GSVA) package v1.42.0; STRING analysis for gene-to-gene and protein-to-protein interaction (http://string-db.org) [123]; Cytoscape v3.9.1 for visualization of protein–protein interaction; CellChat v1.1.3; PCA; UMAP
39	Qing et al. [122]	Takayasu Arteritis (TA)	Chronic	Physiological	PBMC	ScRNA-seq, the Singleron GEXSCOPETM	Seurat v.4.0.1; CellCycleScoring for cell cycle status [124]; FindAllMarkers for DEGs
40	Rajan et al. [125]	The stress of adaptation to different microenvironments	Chronic	Physiological	Osteosarcoma cell lines and tibia and lung metastatic tumors	ScRNA-seq 10× Genomics Chromium	Cell Ranger v3.0.2; Seurat R; silhouette scores for cell similarity evaluation in the same cluster; DESeq2; clusterProfiler; Molecular Signatures Database (MSigDB) Hallmark gene sets; Pseudo-bulk analysis for setting identities of each cell to the sample
41	Reddy et al. [126]	Graft-versus-host incompatibility inflammation	Chronic	Physiological	Intestinal stem cells	ScRNA-seq 10× Genomics Chromium	Seurat R; Cell Ranger in scVI tools; GSEApy package [127] and enrichr API [128] for enrichment pathway analysis; Scanpy; clusterProfiler
42	Reis et al. [129]	Hyperoxia	Chronic	Physiological	Thymi	ScRNA-seq 10× Genomics Chromium	Cell Ranger v2.1.0; PCA & UPMA in the Seurat R v3; edgeR; Ingenuity Pathway Analysis (IPA) for enriched canonical pathway
43	Rodgers et al. [130]	42 days of chronic variable stress (paternal stress)	Chronic	Physical/Psychological	Single-cell zygotes	Single-cell amplification technology on the BioMark HD System using DELTAgene assays	Fluidigm Singular toolset 2.0
44	Romanov et al. [131]	Formalin injection into paw pads	Acute	Physiological/Psychological	Paraventricular nucleus (PVN)	STRT/C1 single cell transcriptomics [34]: C1-AutoPrep system (Fluidigm)	Unbiased clustering analysis; SPIN algorithm [132]
45	Ruden et al. [133]	Hyperosmotic stress (sorbitol), like retinoic acid,	Chronic	Physiological	Mouse embryonic stem cells (mESCs) in vitro culture (recapitulating uterus transplantation)	Bulk and scRNAseq 10× Genomics Chromium	Cell Ranger v6.0.1; Seurat v4.1.1; UMAP
46	Russo et al. [134]	Inflammation and Parkinson’s disease gene LRRK2	Chronic	Physiological/Psychological	Mouse brain microglia cells	ScRNA-seq 10× Genomics Chromium	Seurat; *t*-SNE
47	Salinno et al. [135]	Diabetic disease and in vitro stress	Chronic	Physiological	The Min6 (clone K9) murine β-cell line, EndoC-bH1 human β-cell line, and postnatal day 16 mouse pancreatic islets	ScRNA-seq 10× Genomics Chromium	Louvain clustering; Scanpy v1.4.5.2 or limma v3.38.3 for DEGs; Metascape for Pathway enrichment analysis [71].
48	Shen et al. [136]	Conditioned fear memory	Acute	Physical/Psychological	Hippocampus	ScRNA-seq 10× Genomics Chromium	Seurat R; PCA; *t*-SNE; singleR v1.0.1; DESeq2 v1.2; KEGG pathway enrichment in GSVA R package;
49	Short et al. [137]	Early-life adversity (ELA) and stress	Chronic	Physical/psychological	CRF-expressing hypothalamic paraventricular nucleus	ScRNA-seq SmartSeq2	kallisto [138]; Seurat R; Complex- Heatmap [139]; Metascape
50	Tang et al. [140]	Ischemic acute kidney injury	Acute	Physiological	Kidney	ScRNA-seq	Harmony in Seurat v3.1; NormalizationData, ScaleData, FindClusters for normalizing, scaling data, and clustering [141]; PCA; *t*-SNE; UMPA; FindAllMarker for finding marker genes for clusters
51	Tang et al. [142]	Weaning	Acute	Physiological	Ileum (piglet)	ScRNA-seq 10× Genomics Chromium	Seurat R; *t*-SNE; the shared nearest neighbor (SNN) graph-based method for cell clustering; FindAllMarkers for DEGs in each cell cluster; Monocle 2 v 2.8.0 [120]; CellPhoneDB Python package v2.1.2 for cellular interactions analysis [143]
52	Tatsuoka et al. [144]	Pancreatectomy	Acute	Physical	Islet of pancrease	ScRNA-seq (ddSEQ Single-Cell Isolator (Bio-Rad)) [145]	Seurat R v3.1; PCA and UMAP; CellCycleScoring; Monocle v2.4.0; IPA
53	Tian et al. [146]	Chronic oxidative stress (removal of antioxidants)	Chronic	Physiological	Human iPSC-derived neurons	Genome-wide CRISPR interference and CRISPR activation screens; CROP-seq [147]	Cell Ranger v2.2.0; sgRNA-enrichment libraries [148]
54	Tikhonova et al. [149]	Intraperitoneal administration of fluorouracil (5-FU)-induced hematopoiesis	Acute	Physiological	Cells of the bone marrow niche	ScRNA-seq 10× Genomics Chromium	Cell Ranger; Seurat R [48]; *t*-SNE
55	Vennin et al. [150]	Chronic social defeat stress	Chronic	Psychological	Dorsal and ventral hippocampus	ScRNA-seq 10× Genomics Chromium	Cell Ranger v3.0.2; Seurat v3.1.0; Clustree (v0.4.3) for cluster stability; non-parametric entropy-based Scalable Probabilistic Analysis framework (eSPA) [151]; SingleR v1.2.4; limma package v3.4.4.3; CelltalkDB [152] for Ligand-Receptor Interaction Analysis; mclust R package v5.4.6 for unsupervised clustering
56	Wang et al. [153]	Aging	Chronic	Physiological	Lineage^−^ [Lin^−^] c-Kit^+^ Sca-1^+^ (LKS) hematopoietic stem cells (HSCs) (mouse)	ScRNA-seq SMART-Seq2	smqpp package in Python for pre-processing; STAR; Scanpy v1.7.1; edgeR for cell normalization and logging; Brennecke method for selection of highly variable genes [154]; UMAP and PCA in Scanpy
57	Wang et al. [155]	Acute compartment syndrome (ACS) tibiofibular fractures	Acute	Physical	Deep fascia (fibers, fibroblasts, and immune cells)	ScRNA-seq 10× Genomics Chromium	Cell Ranger v6.1.1; Seurat v3.1.1; PCA; *t*-SNE; FindMarkers for DEGs
58	Wechter et al. [156]	Radiation or etoposide-induced senescence	Acute/chronic	Physiological	Fibroblast cell lines	ScRNA-seq 10× Genomics Chromium	clusterProfiler v4.0.5; Velocyto.py v0.17 for RNA velocity; Seurat
59	Wendorff et al. [157]	Aging	Chronic	Physiological	Hematopoietic stem cells from aging mice	ScRNA-seq 10× Genomics Chromium	Cell Ranger v6.0.2; Seurat v3; Harmony; PCA; UMAP; graphs for visualization in R ggplot2 library
60	Wu et al. [158]	Fibrosis and inflammation	Chronic	Physiological	Kidney tissue of adult Mice	SnRNA-seq using sNuc-DropSeq, DroNc-seq, and 10× Genomics Chromium platforms; Soft Lithography: Used to create silicon masters for DropSeq and DroNc-seq devices	STAR v2.5.3a; Seurat v2.0; CellCycleScoring; canonical correlation analysis [48]; *t*-SNE and cell clustering; FindAllMarkers
61	Wang et al. [159]	Mechanical loading stress	Chronic	Physical	Healthy talus cartilage chondrocytes	ScRNA-seq 10× Genomics Chromium	Cell Ranger v4.0.3; Seurat R v3.1.1; UMAP; Monocle 2; CellChat; STRING v11.0; Cytoscape v3.7.1
62	Wu et al. [158]	Glyphosate toxin	Chronic	Physiological	Liver	ScRNA-seq 10× Genomics Chromium	Cell Ranger v6.0.1; Seurat v4.0.3; PCA; SNN graphs; UMAP; FindAllMarkers; ß CellChat
63	Wen et al. [160]	Rejection of renal transplantation	Chronic	Physiological	Human kidney transplantation biopsy cores	ScRNA-seq databases	Seurat R; Monocle 3; pySCENIC for the transcription factor-centered gene regulatory network [161]
64	Xiao et al. [162]	Stress-induced anxiety	Acute	Psychological	(Mice) Brain tissue-trunk region (S1Tr) and dorsal area (AUD)	ScRNA-seq 10× Genomics Chromium, Spatial transcriptome sequencing	Etho Vision software; Quantity One v4.6.2, (Bio-Rad)
65	Xie et al. [163]	Metabolic stress	Chronic	Physiological	Hematopoietic stem cells	ScRNA-seq (In silico analysis)	Scran; TopHat (v2.1.1) for read mapping; HTSeq (v 0.6.1) for gene counts; EnrichmentMap v2.1.0 in Cytoscape 3.4.0 for visualization of enriched pathways
66	Whitehead and Engler [164]	Aging, myocardial infarction	Chronic	Physiological	Heart tissue of mice	Bulk and scRNA-seq Gene Expression Omnibus database	DESeq2; Graphs of TPM-normalization in R
67	Xu et al. [165]	Stress	Chronic	Physiological	Bulk RNA-seq; 6 tissues (brain, testis, pancreas, esophagus, lung, and spleen) for scRNA-seq	RNA-seq databases; 10× Genomics Visium platform (in silico data analysis)	Seurat v3.9.9; PCA; UMAP; Multi-scale Embedded Gene Co-expression Network Analysis (MEGENA) for gene module identification [166]
68	Xu et al. [167]	Hypoplastic left heart syndrome (HLHS)	Chronic	Physiological	Induced pleuropotent stem cell-cardiomyocytes (iPSC-MC)	ScRNA-seq 10× Genomics Chromium	scds R package v1.6.9; SingleCellNet for cell type classification (v0.1.0) [168]; Seurat; PCA; UMAP; Scanpy; ToppGene [169] for gene enrichment analysis and Gene Ontology; coexpression MSigDB for gene list annotation
69	Yang et al. [170]	Nonalcoholic fatty liver disease	Chronic	Physiological	Liver tissues (human and mouse)	Bulk and scRNA-seq and spatial transcriptomics (in silico analysis)	N.A.
70	Yang et al. [171]	Maternal oocyte aging	Chronic	Physiological	Oocytes	ScRNA-seq databases (In Silico analysis)	DESeq2 v1.32.0; The OmicShare tools (https://www.omicshare.com/) for the GO and KEGG enrichment analysis; the Community Cluster (Glay) algorithm of clusterMaker2 for cell clustering;; STRING; Cytoscape v3.8.2
71	Yanowski et al. [172]	Pancreatectomy	Acute	Physical	Islets of Langerhans (Pancreas)	ScRNA-seq (MARS-seq) [173]	MetaCell analysis for cell clustering [174]; PIC-seq for physical interactions [175]
72	Yoo et al. [176]	Chronic restraint stress	Chronic	Physical/Psychology	Habenula (rat)	the GeneChip Rat Gene 1.0 ST gene microarray (Affymetrix); scRNA-seq (Gene Expression Omnibus Accession No. GSE137478)	Cell Ranger v2; DoubletDecon; Seurat v4 [177]; STRING v11 in Cytoscape v3.8.0
73	Zaleta-Rivera et al. [178]	Restrictive cardiomyopathy	Chronic	Physiological	Ventricular cardiomyocytes of RLC transgenic mice, AAV9-expressing M7.8L shRNA, and nonexpressing shRNA	Single-cell cardiomyocyte calcium transient traces using the IonOptix Myocyte Calcium and Contractility Recording System	N.A.
74	Yu et al. [179]	Interstitial cystitis/bladder pain syndrome (IC/BPS) by hydrochloride instillation	Chronic	Physiological	Bladder	Two-photon intravital imaging and single-cell microarray transcriptome analysis [180]	Metacore; GSEA
75	Yu et al. [181]	Transplantation, inflammation, and genotoxic stress	Chronic	Physical/Physiological	Hematopoietic stem cells (HSCs)	SMART-seq2 for scRNA-seq	PAGODA package v1.99.3 [182]
76	Zadora et al. [183]	Preeclampsia	Chronic	Physiological	Preeclamptic placentas of human, mouse, and monkey	Omnibus datasets	IPA; 7500 Fast System Software (Applied Biosystems); PrimerExpress 3.0 (Applied Biosystems)
77	Zaman et al. [184]	Hypertension induced by angiotensin III	Acute and Chronic	Physical	cardiac resident macrophages (cardiomuscular tissue/cardiomyocytes)	ScRNA-seq 10× Genomics Chromium	Cell Ranger; hashtag antibody barcoding libraries [185]; MULTI-seq algorithm for decomplexing of cells; SNN graph-based clustering; UMAP visualization; Garnett machine learning algorithm for gene signatures; Seurat v.3.1; SCTransform; gProfiler; Harmony
78	Zhang et al. [186]	Diabetic nephropathy	Chronic	Physiological	OXPHOS chain in diabetic kidney tissue, PLEKHA1 gene	The Gene Expression Omnibus database (in silico analysis)	Weighted gene co-expression network analysis (WGCNA) for DEGs and co-expression gene modules [98]; XGBoost for prediction models and K trees for patient risk prediction [187]; Least absolute shrinkage and selection operator (LASSO) in mixOmics for identification of diagnostic gene sets [188,189], CIBERSORT deconvolution algorithm for immune cell abundance estimation [190]
79	Zhang et al. [191]	Aortic aneurysm and dissection (AAD)	Chronic	Physical	Thoracic aortic walls of wild-type mice	ScRNA-seq & scATAC-seq 10× Genomics	SNN graphs; FindMarkers; AddModuleScore in Seurat v3.0.0 for the gene list in each cluster; WaVE-EdgeR for DGEs [192]; clusterProfiler for Gene Ontology (GO) analysis [193]
80	Zhang et al. [194]	Ultra-small nanoclusters (USNCs, <2 nm)	Chronic	Physical	PBMC	Single-cell mass cytometry and magnetic luminex assay	xCell; Cytobank

## Data Availability

No new data were created or analyzed in this study.

## Supplementary Materials

The following supporting information can be downloaded at: https://www.mdpi.com/article/10.3390/cells15090807/s1, Method S1.
