# Supplementary material for: Mosaic: Single-Cell Atlas of Stress
Source: Cells. 2026 Apr 29;15(9):807. doi: 10.3390/cells15090807 (PMC13163097; doi:10.3390/cells15090807)
Supplement: Supplementary file 1 [file cells-15-00807-s001.zip › cells-4198296-supplementary.pdf]

Supplementary Method S1

PubMed. Coverage: 1946 – current. First searched on September 5, 2023. Updated on January 26, 2026. 2309 results total.

(("single-cell analysis"[MeSH terms] OR "single-cell analysis"[Title/Abstract] OR "single-cell analyses"[Title/Abstract] OR "single-cell omics"[Title/Abstract] OR "single-cell multi-omics"[Title/Abstract] OR "single-cell multiomics"[Title/Abstract]) OR ("single-cell"[Title/Abstract] OR "single cells"[Title/Abstract] OR "individual cell"[Title/Abstract] OR "individual cells"[Title/Abstract]) AND ("epigenom*"[Title/Abstract] OR "epitranscriptom*"[Title/Abstract] OR "genomics"[MeSH terms] OR "genom*"[Title/Abstract] OR "metabolomics"[MeSH terms] OR "metabolom*"[Title/Abstract] OR "phenomics"[MeSH terms] OR "phenome"[Title/Abstract] OR "phenomes"[Title/Abstract] OR "phenomic*"[Title/Abstract] OR "proteom*"[Title/Abstract] OR "gene expression profiling"[MeSH terms] OR "gene expression analys*"[Title/Abstract] OR "gene expression monitor*"[Title/Abstract] OR "gene expression profil*"[Title/Abstract] OR "transcript expression analys*"[Title/Abstract] OR "transcriptom*"[Title/Abstract])) AND ("stress, physiological"[MeSH] OR "stress, psychological"[MeSH terms] OR "stress"[Title/Abstract] OR "stresses"[Title/Abstract] OR "stressor"[Title/Abstract] OR "stressors"[Title/Abstract])

No limits were used.

CINAHL via EBSCOhost. Coverage: 1937 – current. Searched on September 5, 2023. Updated on January 26, 2026. 64 results total.

((TI "single-cell analysis" OR AB "single-cell analysis" OR TI "single-cell analyses" OR AB "single-cell analyses" OR TI "single-cell omics" OR AB "single-cell omics" OR TI "single-cell multi-omics" OR AB "single-cell multi-omics" OR TI "single-cell multiomics"

OR AB "single-cell multiomics") OR (TI "single-cell" OR AB "single-cell" OR TI "single cells" OR AB "single cells" OR TI "individual cell" OR AB "individual cell" OR TI "individual cells" OR AB "individual cells") AND (TI "epigenom*" OR AB "epigenom*" OR TI "epitranscriptom*" OR AB "epitranscriptom*" OR MH "genomics+" OR TI "genom*" OR AB "genom*" OR MH "metabolomics" OR TI "metabolom*" OR AB "metabolom*" OR TI "phenome" OR AB "phenome" OR TI "phenomes" OR AB "phenomes" OR TI "phenomic*" OR AB "phenomic*" OR TI "proteom*" OR AB "proteom*" OR MH "gene expression profiling" OR TI "gene expression analys*" OR AB "gene expression analys*" OR TI "gene expression monitor*" OR AB "gene expression monitor*" OR TI "gene expression profil*" OR AB "gene expression profil*" OR TI "transcript expression analys*" OR AB "transcript expression analys*" OR TI "transcriptom*" OR AB "transcriptom*")) AND (MH "stress+" OR MH "stress, physiological" OR TI "stress" OR AB "stress" OR TI "stresses" OR AB "stresses" OR TI "stressor" OR AB "stressor" OR TI "stressors" OR AB "stressors")

No limits were used.

PsycInfo via EBSCOhost. Coverage: 1800 – current. Searched on September 5, 2023. Updated on January 26, 2026. 55 results total.

((TI "single-cell analysis" OR AB "single-cell analysis" OR TI "single-cell analyses" OR AB "single-cell analyses" OR TI "single-cell omics" OR AB "single-cell omics" OR TI "single-cell multi-omics" OR AB "single-cell multi-omics" OR TI "single-cell multiomics" OR AB "single-cell multiomics") OR (TI "single-cell" OR AB "single-cell" OR TI "single cells" OR AB "single cells" OR TI "individual cell" OR AB "individual cell" OR TI "individual cells" OR AB "individual cells") AND (TI "epigenom*" OR AB "epigenom*" OR TI "epitranscriptom*" OR AB "epitranscriptom*" OR MH "genomics+" OR TI "genom*" OR AB "genom*" OR TI "metabolom*" OR AB "metabolom*" OR TI "phenome" OR AB "phenome" OR TI "phenomes" OR AB "phenomes" OR TI "phenomic*" OR AB "phenomic*" OR TI "proteom*" OR AB "proteom*" OR TI "gene expression analys*" OR AB "gene expression analys*" OR TI "gene expression monitor*" OR AB "gene expression monitor*" OR TI "gene expression profil*" OR AB "gene expression profil*" OR TI "transcript expression analys*" OR AB "transcript expression analys*" OR TI "transcriptom*" OR AB "transcriptom*")) AND (MH "stress+" OR TI "stress" OR AB "stress" OR TI "stresses" OR AB "stresses" OR TI "stressor" OR AB "stressor" OR TI "stressors" OR AB "stressors")

No limits were used.

Scopus. Coverage: 1996 – current. Searched on September 5, 2023. Updated on January 26, 2026. 838 results total.

((TITLE-ABS-KEY ("single-cell analysis") OR TITLE-ABS-KEY ("single-cell analyses") OR TITLE-ABS-KEY ("single-cell omics") OR TITLE-ABS-KEY ("single-cell multi-omics") OR TITLE-ABS-KEY ("single-cell multiomics")) OR (TITLE-ABS-KEY ("single-cell") OR TITLE-ABS-KEY ("single cells") OR TITLE-ABS-KEY ("individual cell") OR TITLE-ABS-KEY ("individual cells")) AND (TITLE-ABS-KEY (epigenom*) OR TITLE-ABS-KEY (epitranscriptom*) OR TITLE-ABS-KEY (genom*) OR TITLE-ABS-KEY (metabolom*) OR TITLE-ABS-KEY (phenome) OR TITLE-ABS-KEY (phenomes) OR TITLE-ABS-KEY (phenomic*) OR TITLE-ABS-KEY (proteom*) OR TITLE-ABS-KEY ("gene expression analys*") OR TITLE-ABS-KEY ("gene expression monitor*") OR TITLE-ABS-KEY ("gene expression profil*") OR TITLE-ABS-KEY ("transcript expression analys*") OR TITLE-ABS-KEY (transcriptom*))) AND (TITLE-ABS-KEY (stress) OR TITLE-ABS-KEY (stresses) OR TITLE-ABS-KEY (stressor) OR TITLE-ABS-KEY (stressors)) AND NOT INDEX (medline)

This search excludes MEDLINE results because PubMed (above) includes MEDLINE. No other limits were used.
